# Ask1 and Akt act synergistically to promote ROS-dependent regeneration in *Drosophila*

**DOI:** 10.1371/journal.pgen.1007926

**Published:** 2019-01-24

**Authors:** Paula Santabárbara-Ruiz, José Esteban-Collado, Lidia Pérez, Giacomo Viola, Josep F. Abril, Marco Milán, Montserrat Corominas, Florenci Serras

**Affiliations:** 1 Department of Genetics, Microbiology and Statistics, School of Biology and Institute of Biomedicine of the University of Barcelona (IBUB), University of Barcelona, Barcelona, Spain; 2 Institute for Research in Biomedicine (IRB Barcelona), The Barcelona Institute of Science and Technology, Baldiri Reixac, Barcelona, Spain; 3 Institució Catalana de Recerca i Estudis Avançats (ICREA), Pg. Lluís Companys, Barcelona, Spain; The University of North Carolina at Chapel Hill, UNITED STATES

## Abstract

How cells communicate to initiate a regenerative response after damage has captivated scientists during the last few decades. It is known that one of the main signals emanating from injured cells is the Reactive Oxygen Species (ROS), which propagate to the surrounding tissue to trigger the replacement of the missing cells. However, the link between ROS production and the activation of regenerative signaling pathways is not yet fully understood. We describe here the non-autonomous ROS sensing mechanism by which living cells launch their regenerative program. To this aim, we used *Drosophila* imaginal discs as a model system due to its well-characterized regenerative ability after injury or cell death. We genetically-induced cell death and found that the Apoptosis signal-regulating kinase 1 (Ask1) is essential for regenerative growth. Ask1 senses ROS both in dying and living cells, but its activation is selectively attenuated in living cells by Akt1, the core kinase component of the insulin/insulin-like growth factor pathway. Akt1 phosphorylates Ask1 in a secondary site outside the kinase domain, which attenuates its activity. This modulation of Ask1 activity results in moderate levels of JNK signaling in the living tissue, as well as in activation of p38 signaling, both pathways required to turn on the regenerative response. Our findings demonstrate a non-autonomous activation of a ROS sensing mechanism by Ask1 and Akt1 to replace the missing tissue after damage. Collectively, these results provide the basis for understanding the molecular mechanism of communication between dying and living cells that triggers regeneration.

## Author summary

One of the early events that occur after tissue damage consists on the production of Reactive Oxygen Species (ROS), that signal to the surrounding tissue to initiate wound healing and regeneration. Many signaling pathways, such as JNK and p38, respond to oxidative stress and are necessary for regenerative growth. As the link between ROS and regenerative signaling is not well understood, we decided to explore the mechanism that underlies this process. To do that, we genetically induced cell death in specific areas of *Drosophila* wing imaginal discs and then studied the mechanism that drives living cells to replace the damage zone until it is completely regenerated. We found that the *Drosophila* Apoptosis signal-regulating kinase 1 (Ask1), a protein that is sensitive to oxidative stress, is a key player in this scenario. This protein acts as an intracellular sensor that upon damage activates the JNK and p38 regenerative signaling pathways. However, high activity of Ask1 can be toxic for the cell. This is controlled by Akt, a kinase downstream the insulin pathway, which attenuates the activity of Ask1 in the living cells that will participate in the regeneration process. In consequence, Ask1 and Akt act synergistically to respond to the stress generated after tissue damage and drive regeneration. Our results provide a first overview within the framework of how insulin signaling inputs could modulate the capacity to overcome tissue damage.

## Introduction

Organisms are continuously exposed to a wide variety of environmental stressors that can cause deterioration and cell death. Tissues overcome the effect of those stressors by replacing damaged cells to restore homeostasis. Therefore, understanding the early signals that initiate the response to damage is an essential issue in regenerative medicine. Regeneration can be monitored in *Drosophila* imaginal discs, which are well-characterized epithelial sacs capable to regenerate after genetically-induced apoptosis or when parts are physically removed (reviewed in [1]). Compiling evidence supports that reactive oxygen species (ROS) fuel wound healing and oxygen-dependent redox-sensitive signaling processes involved in damage response [2–4]. Actually, genetically-induced apoptosis in the imaginal discs, using the Gal4/UAS system, leads to the production of ROS which propagate to the surrounding neighbors [5–7]. Although oxidative stress has been associated with several pathologies, it has been described that low levels of ROS can be beneficial for signal transduction [8].

The Jun-N Terminal kinase (JNK) and p38 signaling pathways are MAP kinases that respond to many stressors, including ROS, and foster regeneration and cytokine production in *Drosophila* [5,6,16–25,7,26,27,9–15]. Both pathways control numerous cellular processes as diverse as cell proliferation and cell death. For example, ectopic activation of JNK induces apoptosis [28,29], but its inhibition results in lethality [30]. It is though that these disparities could be due to either different levels of activity or different mechanisms of activation.

After genetic ablation in a specific zone of the wing imaginal disc, dying cells produce high levels of ROS, and show high JNK activity [5,7]. High levels of JNK have been associated to dying cells and are involved in a feedback amplification loop to enhance the apoptotic response to stress [31]. However, the living cells nearby the dying zone show low levels of ROS, which are beneficial for the cell as they turn on the activation of p38 and low levels of JNK [5]. Both MAP kinases in the living cells are required for a cytokine-dependent regenerative growth. A key question is how the balance between the beneficial or detrimental effects of ROS is controlled and, in particular, how ROS control JNK and p38 activity. A candidate molecule to perform this function is the MAPKKK Apoptosis signal-regulating kinase 1 (Ask1), which responds to various stresses by phosphorylation of the kinases upstream JNK and p38 [32–34]. Hence, in a reduced environment, thioredoxin (Trx) inhibits Ask1 kinase activity by directly binding to the N-terminal region of Ask1. Upon oxidative stress, the redox-sensitive cysteines of Trx become oxidized, resulting in the dissociation of Trx from Ask1. Consequently, Ask1 is oligomerized and its threonine-rich kinase domain is phosphorylated, inducing Ask1 activation [35,36]. Mammalian Ask1 is highly sensitive to oxidative stress and contributes substantially to JNK-dependent apoptosis. Nevertheless, recent studies have also revealed other functions of this kinase, including cell differentiation and survival [37].

Ask1-interacting proteins promote conformational changes that lead to the modulation of Ask1 activity and result in various cellular responses. For example, Ask1 is a substrate for phosphorylation by Akt1, a serine/threonine kinase activated by lipid kinase phosphatidylinositide 3-kinase (Pi3K) pathway in response to insulin receptor activation. This phosphorylation is associated to a decrease of Ask1 activity *in vitro* [38]. The Pi3K/Akt pathway, which is conserved between mammals and *Drosophila*, is one of the main effector signals for the regulation of tissue growth [39–41]. In *Drosophila*, loss-of–function mutants of various components of the pathway result in reduced body size or lethality [42]. Conversely, mutants of the phosphatase *PTEN*, an antagonist of Pi3K, result in high activity of Akt and tissue overgrowth [43–45]. Thus, it is conceivable that Pi3K/Akt signaling is involved in regenerative growth, but whether Pi3K/Akt and Ask1 interact for controlling regeneration is unknown and deserves attention.

In this work, we genetically induced cell death in wing imaginal discs to explore the link between ROS production and regeneration. We found that Ask1 acts as a sensor of ROS upstream the JNK and p38 pathways. Moreover, we describe that Akt1 is necessary for modulating Ask1 activity in living cells to trigger regeneration. In addition, our results indicate that oxidative stress generated in the damaged cells signals the neighboring living cells to promote tissue repair.

## Results

### Ask1 is necessary for regenerative growth

**Fig 1:**
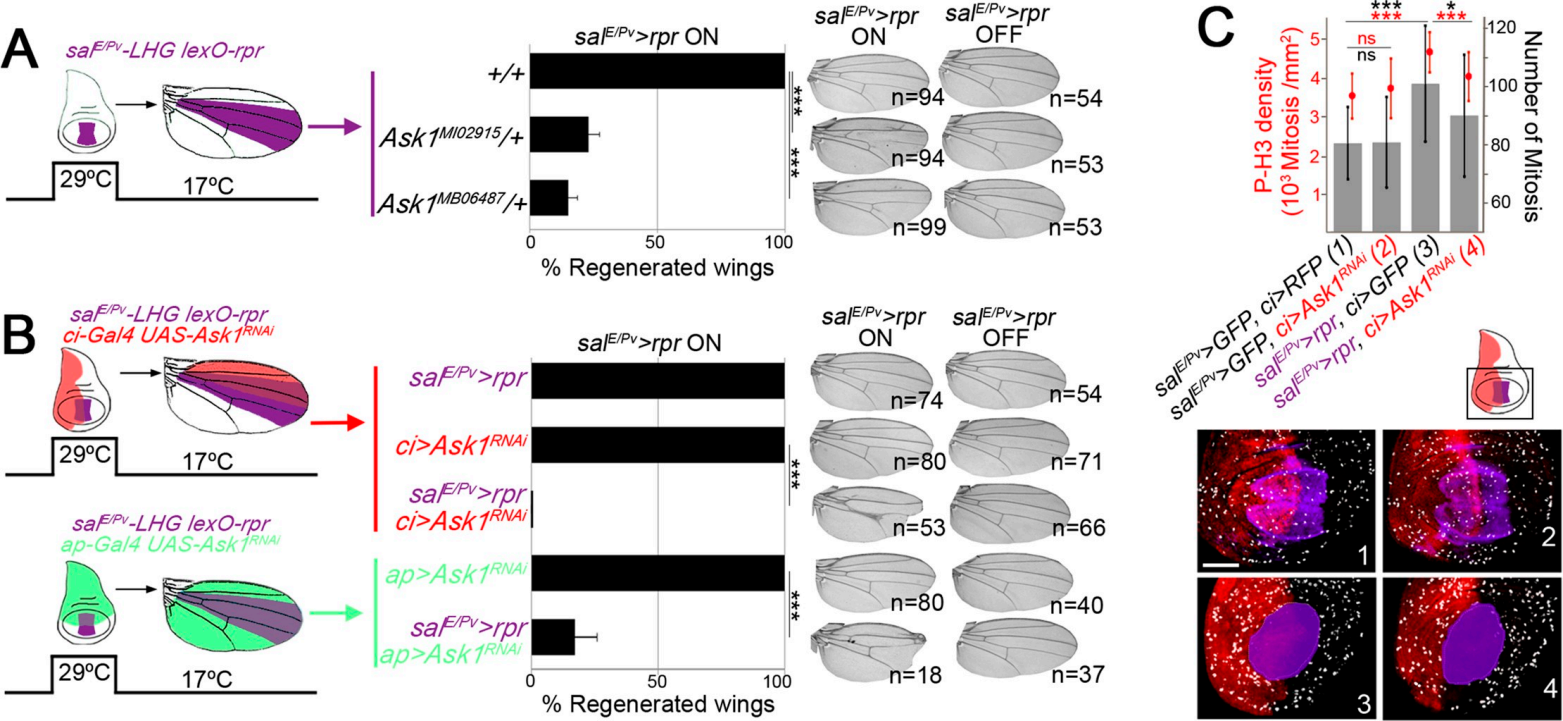
Ask1 is necessary for imaginal disc regeneration. (A) Scheme of the zone ablated (purple) in a wing imaginal disc and its corresponding region in the adult wing (left). Percentage of fully regenerated wings (normal set of veins and interveins) in wild type and *Ask1* mutant backgrounds after genetic ablation. On the right, examples of wings resulting after *rpr* activation (*sal*^*E/Pv*^*>rpr* ON; developed at 17°C and for 11hours at 29°C on day 8^th^ after egg laying) and wings control, in the absence of cell death but with the same genotype (*sal*^*E/Pv*^*>rpr* OFF, kept at 17°C from egg to adult) for the genotypes indicated. The number of cases analyzed in both conditions is indicated. (B) Double transgene activation scheme. The zone ablated in the wing disc using *sal*^*E/Pv*^*LHG lexO-rpr* and its corresponding region in the adult wing are indicated in purple; the zone of the *Ask1*^*RNAi*^ transgene expression is indicated in red or green. Percentage of normal wings (*Ask1*^*RNAi*^ expression alone), fully regenerated wings (*rpr* expression alone) and experimental samples (*rpr* and *Ask1*^*RNAi*^ dual expression). On the right, examples of resulting wings with *rpr* ON or OFF and the number of cases analyzed for each condition. (C) Phosphorylated H3 as indicator of mitotic cells per area (red) and number of mitosis (black) in discs with the dual transactivation system using *rpr* and *Ask1*^*RNAi*^ for the genotypes indicated (n = 30 for each genotype). The black square in the cartoon indicates the area used for analysis; below, examples of discs for the four genotypes used in this experiment. Note that the images have been colorized to maintain uniformity. Purple: GFP (1 and 2), cell death (3 and 4); Red: RFP (1), GFP (3), anti-Ci (2, 4). Mitosis (P-H3) in white. Bar: 50μm. Error bars in (A,B) show standard error of sample proportion and in (C) standard deviation. ***P<0.001.

The Gal4/UAS/Gal80^TS^ transactivation system has been extensively used to temporarily and spatially activate pro-apoptotic genes such as *reaper* (*rpr*) [10,11,46]. To study the capacity to regenerate, we induced cell death in the wing disc using the wing-specific *sal*^*E/Pv*^-*Gal4* strain to activate *UAS-rpr*, in a temporally controlled manner thanks to the *tub-Gal80*^*TS*^ thermo sensitive Gal4-repressor (henceforth *sal*^*E/Pv*^*>rpr*). We first kept the embryos at 17°C until the 8th day (192 hours after egg laying) to activate the *tub-Gal80*^*TS*^ and therefore to prevent *rpr* expression. Subsequently, we moved those larvae to 29°C for 11 hours, to inhibit *tub-Gal80*^*TS*^, and activate apoptosis in the *sal*^*E/Pv*^-*Gal4* zone. Then, larvae were returned to 17°C to avoid further apoptosis and allow tissue to regenerate. After adults emerged, wings were dissected and regeneration was analyzed. With these experimental conditions, 100% of the wings of *sal*^*E/Pv*^*>rpr* flies contained the full set of veins and interveins, which demonstrates that the missing parts were regenerated (Fig 1A).

The same experiment was carried out in *Ask1* mutant backgrounds. The *Ask1*^*MB06487*^ mutant flies are viable in homozygosis but lethal over the deficiency *Df(3R)BSC636*, which suggests that is a hypomorphic allele. The *Ask1*^*MI02915*^ mutant is lethal in homozygosis as well as over the *Df(3R)BSC636*, which suggests that is an amorphic allele. We found that after genetically-induced cell death in *Ask1*^*MB06487*^ or *Ask1*^*MI02915*^ heterozygous background, full regeneration is only achieved in 15% and 23% of the wings, respectively (Fig 1A). The phenotypes of the abnormally regenerating wings vary and consisted in lack of some veins or parts of a vein or anomalous vein segments (S1A Fig).

To further address the role of *Ask1* in regeneration, we used a double transactivation system to simultaneously induce cell death in the *sal*^*E/Pv*^ domain and inhibit *Ask1* with *UAS-RNAi* (*UAS-Ask1*^*RNAi*^) in an adjacent compartment (Fig 1B). RNAi knockdown of *Ask1 (UAS-Ask1*^*RNAi*^) reduces *Ask1* mRNA total levels to approximately 25–30 percent of that observed in controls (S1B Fig). The *UAS-Ask1*^*RNAi*^ transgene was activated in the anterior compartment using the Gal4/UAS system (*ci-Gal4>UAS-Ask1*^*RNAi*^) and cell death was induced in the *sal*^*E/Pv*^ domain using the Gal80-repressible transactivator system *LHG* (*L**exA-**H**inge-**G**al4* activation domain), a modified form of the *lexA lexO* system (*sal*^*E/Pv*^*-LHG>lexO-rpr*) (Fig 1B) [5,47]. Both transgenes were activated at the same time, following the same protocol as in the previous experiment. The resulting adult wings (*ci>Ask1*^*RNAi*^ *sal*^*E/Pv*^*>rpr*) lacked some veins or interveins and their size was reduced in all cases observed. However, the inhibition of *Ask1* in the anterior compartment for the same time but without cell death, did not affect vein pattern nor wing size (*ci>Ask1*^*RNAi*^) (Fig 1B). Similar results were obtained after driving *UAS-Ask1*^*RNAi*^ to a different compartment (dorsal) and inducing cell death in *sal*^*E/Pv*^ (*ap>Ask1*^*RNAi*^ *sal*^*E/Pv*^*>rpr*); in this case, we found that 82% of wings could not regenerate properly. As for the anterior compartment, *Ask1*^*RNAi*^ in the dorsal compartment without cell death did not affect vein pattern nor wing size (*ap>Ask1*^*RNAi*^) (Fig 1B). To discard any toxicity due to the insertion of the transgene, all experiments were carried out in parallel but constantly at 17°C to maintain *tub-Gal80*^*TS*^ activity and block transgene (*UAS-* or *lexO-*) expression. In these conditions no defects in the wings were detected (*sal*^*E/Pv*^> OFF in Fig 1A and 1B).

To evaluate whether these anomalies were due to impairment of proliferation, we analyzed the mitotic index, calculated as the number of cells positive for the phosphorylated form of Histone 3 (P-H3) in the anterior compartment (*ci>*), where the *Ask1*^*RNAi*^ transgene was expressed. It is well known that apoptosis in discs induces compensatory proliferation of nearby cells (reviewed in [1]). Accordingly, genetic ablation (*sal*^*E/Pv*^*>rpr ci>GFP*) resulted in an increase of mitosis compared to the neutral (*sal*^*E/Pv*^*>GFP ci>RFP*) or to the *Ask1* knockdown (*sal*^*E/Pv*^*>GFP ci>Ask1*^*RNAi*^) conditions. However, combining genetic ablation in the *sal*^*E/Pv*^ zone with *Ask1* knockdown in the nearby anterior compartment, the number of mitosis associated to damage did not increase (*sal*^*E/Pv*^*>rpr ci>Ask1*^*RNAi*^) (Fig 1C). This observation confirms that *Ask1* reduction impairs regenerative growth.

### Ask1 activity is higher in apoptotic cells than in regenerating cells

**Fig 2:**
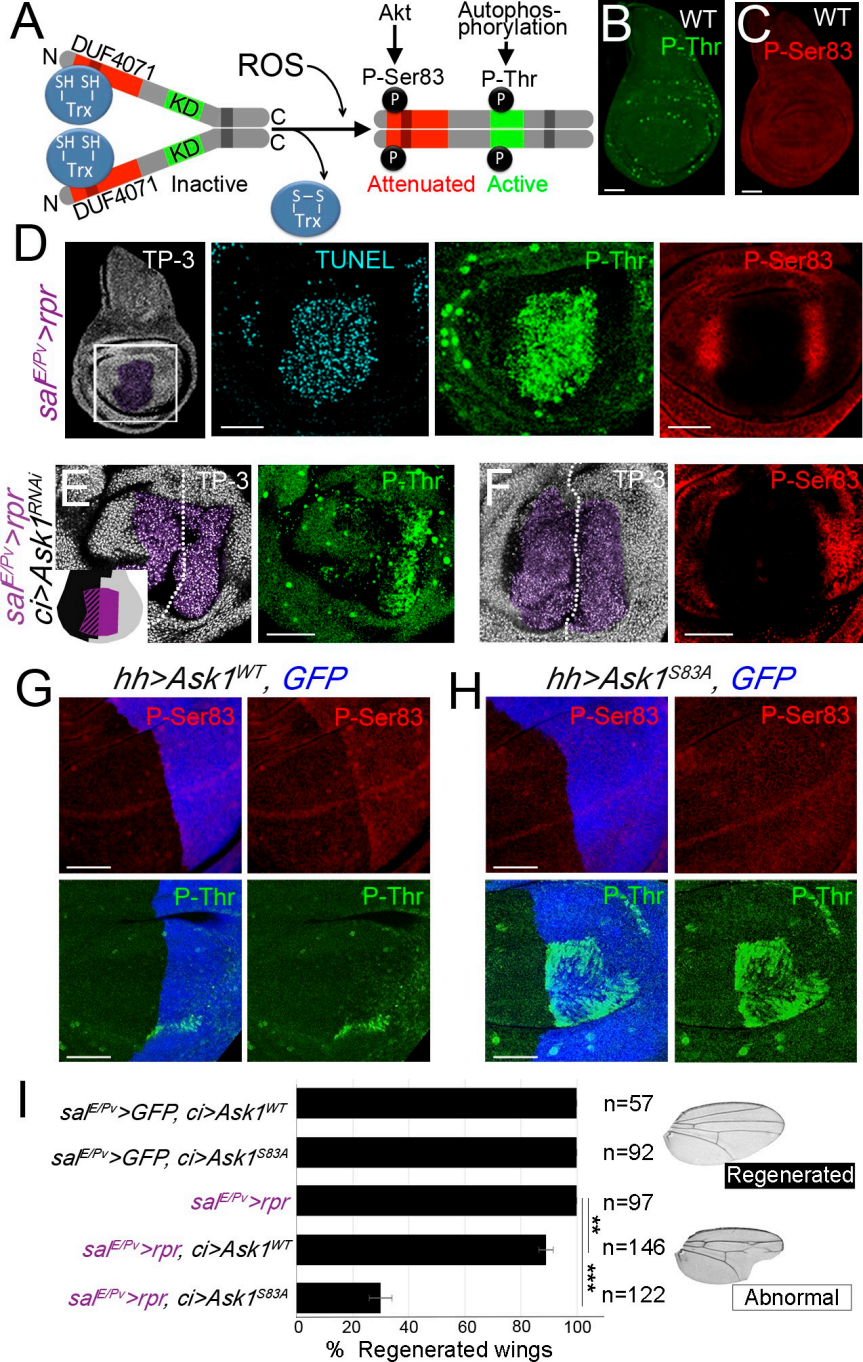
Ask1 activation in apoptotic cells and in regenerating cells. (A) Structural features of Ask1-RC isoform. The phosphokinase domain (KD) with a highly conserved core of threonines is shown in green. The DUF4071 domain, close to the N-terminal is shown in red. Inactive Ask1 is constituted by association of Ask1 monomers through the coiled coil domain (grey shadow) and the reduced form of thioredoxin (Trx-SH) in the N-terminal domain (left). Upon oxidation, Trx-S-S dissociates, which allows Ask1 autophosphorylation in the phosphokinase domain, leading to an active Ask1. The C-terminal coiled coil domain (dark grey) allows interactions between Ask1 monomers to form oligomers. Active Akt phosphorylates Ask1 on Ser83 residue. (B-C) Characterization of mammalian P-ASK1 antibodies in *Drosophila* discs. Endogenous levels of P-Thr (B) and P-Ser83 (C) in wild-type (WT) discs. (D) Ask1 activity in *sal*^*E/Pv*^*>rpr* discs. *Sal*^*E/Pv*^*>rpr* disc indicating the area imaged (white square); TP-3 (TO-PRO-3) nuclei; purple area: the *sal*^*E/Pv*^*>rpr* ablated zone. Disc stained with TUNEL assay and Ask1 P-Thr, (n = 21). Disc stained with Ask1 P-Ser83 (n = 35). (E-F) After cell death (purple) and simultaneous inhibition of *Ask1* in the anterior compartment (*sal*^*E/Pv*^*>rpr ci>Ask1*^*RNAi*^), P-Thr (E) and P-Ser83 (F). White-dotted line indicates the AP boundary; anterior to the left, posterior to the right. The inset in (E) depicts the *Ask1*^*RNAi*^ area (black) and the *sal*^*E/Pv*^*>rpr* ablated zone (purple). (G) Ectopic expression of the *Ask1*^*WT*^ form in the posterior compartment (blue). (H) Ectopic expression of the mutant form *Ask1*^*S83A*^. Scale bars 50μm. (I) Percentage of regenerated wings in controls (*rpr*, *Ask1*^*WT*^ *or Ask1*^*S83A*^ expression alone) and experimental samples (*rpr* and *Ask1*^*WT*^ *or Ask1*^*S83A*^ dual expression). Right: Examples of a wing with normal and abnormal regeneration (*sal*^*E/Pv*^*>rpr*, *ci> Ask1*^*S83A*^). Error bars show standard error of sample proportion. **P<0.01 ***P<0.001.

The *Drosophila Ask1* locus encodes two protein isoforms of different lengths, Ask1-RB and Ask1-RC, with predicted molecular weights of 136.5 kDa and 155.5 kDa, respectively. Both have a protein kinase-like domain that contains a highly conserved core of threonines conferring functionality to the protein (reviewed in [32]). It has been reported that after Trx release, the Ask1 oligomer undergoes conformational change leading to trans-autophosphorylation of the threonine residue corresponding to Thr838, Thr845 and Thr747 in human, mouse and *Drosophila* Ask1, respectively [32]. In human cells, phosphorylation of the N-terminus Ser83 by Akt results in attenuation of Ask1 activity *in vitro* [38]. This is noteworthy, because attenuation of Ask1 could result in moderate levels of activity necessary for turning on JNK and p38 in living cells. *Drosophila* Ask1 lacks this residue and the Akt consensus motif in the N-terminus (S2 Fig, S1 Appendix, S2 Appendix). However, we found that the longer Ask1-RC isoform contained a conserved domain of unknown function DUF4071 with a highly conserved Ser (human Ser174, *Drosophila* Ser83), which we hypothesized that could be key in Ask1 regulation (Fig 2A, S2 Fig).

Active Ask1 can be traced with antibodies against the phosphorylated threonine residues of the highly conserved kinase domain (henceforth P-Thr) [48] and the attenuated form with specific antibodies against phosphorylated Ser83 (P-Ser83). We first tested both antibodies in wild-type wing imaginal discs. We detected low levels of P-Thr all over the disc including some transient high activity during mitosis (Fig 2B and S3A Fig), as occurs in dividing mammalian cells [49]. In *Ask1* mutant backgrounds, both the general and the mitosis-associated P-Thr levels were reduced but not abolished (S3B Fig), and *Ask1*^*RNA*i^ discs did not show any effect on P-Thr levels in mitosis (Fig 2E). This discrepancy may be due to the hypomorphic condition of the mutant combinations, but also to that the antibody may recognize some unspecific epitope in mitotic cells. We also found basal levels of P-Ser83 in wild-type discs (Fig 2C). Ectopic expression of *Ask1*^*RNA*i^ suppressed the low endogenous P-Ser83 Ask1 levels (S3C Fig).

Remarkably, upon apoptosis, high levels of P-Thr were localized in dying cells (Fig 2D) and absent or, in some cases, increased weakly above the basal levels in nearby living cells. This increase of P-Thr in apoptotic cells was inhibited after *Ask1*^*RNAi*^ expression (Fig 2E). In contrast, P-Ser83 accumulated in living cells adjoining the apoptotic zone and was completely absent in apoptotic cells. The increase in P-Ser83 varied from discs with strong accumulation near the dying domain to those with an extended increase in the whole wing pouch (2D and S4A Figs). Similar results were obtained after killing cells with a different pro-apoptotic gene (*sal*^*E/Pv*^*>hid*) (S4B Fig). In the presence of apoptosis and blocking *Ask1*, the P-Ser83 increase in living cells was inhibited (*sal*^*E/Pv*^*>rpr ci>Ask1*^*RNAi*^, Fig 2F). Moreover, P-Ser83 was also found to be elevated at the wound edges after physical injury (S4C Fig). Together, these observations indicate that living cells respond to damage by phosphorylation of Ask1 Ser83.

In contrast to the high activity of Ask1 in dying cells (high P-Thr), the presence of P-Ser83 in living cells could be indicative of tolerable levels of Ask1 achieved by attenuation of P-Thr activity. To test this hypothesis, we mutated *Ask1* at serine 83 to alanine and cloned it into a UAS vector (*UAS-Ask1*^*S83A*^). In parallel, we also cloned a wild-type form of *Ask1* (*UAS-Ask1*^*WT*^). We ectopically expressed *Ask1*^*WT*^ in the posterior compartment of the wing disc (*hh-Gal4 UAS-Ask1*^*WT*^), which resulted in an increase of P-Ser83, in addition to low levels of P-Thr (Fig 2G). Interestingly, the ectopic expression of *Ask1*^*S83A*^ in the posterior compartment (*hh-Gal4 UAS-Ask1*^*S83A*^), did not show a rise in P-Ser83, but strong activation of P-Thr (Fig 2H). The high levels of P-Thr were not homogeneously distributed, varied from disc to disc, and were always found within the posterior compartment *(hh>)*, where the transgene was activated. This observation concurs with the P-Ser83 residue as responsible for the attenuation of P-Thr.

We next analyzed whether the Ser83 residue was key for the damage response. Using the double transactivation system, we found that the expression of *UAS-Ask1*^*WT*^ or *UAS-Ask1*^*S83A*^ transgenes in the anterior compartment without cell death did not cause any defects in wing morphology, size or vein and intervein patterning (*sal*^*E/Pv*^*>GFP ci>Ask1*^*WT*^ or *Ask1*^*S83A*^ in Fig 2I). After genetic ablation, expression of *UAS-Ask1*^*WT*^ resulted in full regeneration in 89% of wings *(sal*^*E/Pv*^*>rpr ci>Ask1*^*WT*^*)*. In contrast, expression of *UAS-Ask1*^*S83A*^ led to a fall to only 29% of individuals being capable of regenerate and the rest showed strong effects on wing morphology, with altered pattern of veins and interveins, as well as the appearance of notches, which together are indicative of disrupted regeneration *(sal*^*E/Pv*^*>rpr ci >Ask1*^*S83A*^ in Fig 2I). The suppression of the ability to regenerate by *UAS-Ask1*^*S83A*^, which may act as a dominant negative allele, is likely due to the lack of non-autonomous P-Ser83 increase in the living cells near the damaged zone.

### Pi3K/Akt signaling attenuates Ask1 activity in living cells

Next, we decided to identify the upstream signal responsible for Ser83 phosphorylation. In mammalian cells, attenuating phosphorylation of Ask1 is driven by the serine-threonine Akt kinase, the core kinase of the insulin pathway [38]. We wondered whether the *Drosophila* Akt1 as well as its upstream Pi3K92E kinase (the *Drosophila* Pi3K kinase also known as dp110), were required for Ser83 phosphorylation.

**Fig 3:**
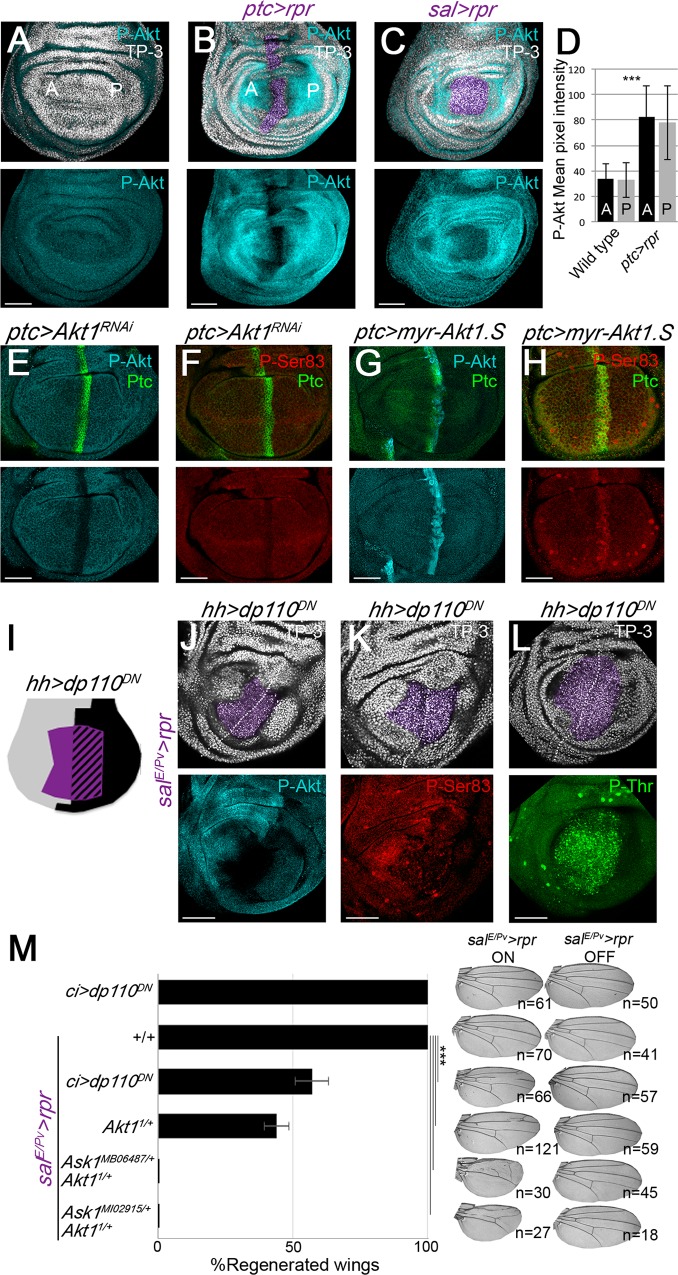
Phosphorylation of *Drosophila* Ask1 Ser83 depends on the Pi3K/Akt1 pathway. (A-C) P-Akt staining in wild-type discs (A), and after *ptc>rpr* (B), and *sal>rpr* induction (C). Apoptotic zone (pyknotic nuclei) in purple. (D) Mean pixel intensity for the wild type and *ptc>rpr* taken from regions of interest in the A and P compartments as shown in the images. (E,F) RNA interference of *Akt1* in the *ptc* stripe of cells (*ptc>Akt1*^*RNAi*^) and (G,H) ectopic expression of a constitutively active form of *Akt1* (*ptc>myr-Akt1*.*S*), stained with P-Akt and Ask1 P-Ser83. (I) Design of the experiments in J,K and L with *sal*^*E/Pv*^*>rpr* induction (purple) and blocking Pi3K (*hh>dp110*^*DN*^) in the posterior compartment (black). Staining with anti-P-Akt (J), with anti-Ask1 P-Ser83 (K) with Ask1 P-Thr (L). White-dotted line indicates anterior-posterior boundary. TP-3; TO-PRO-3 nuclear staining. Scale bars 50μm. (M) Percentage of fully regenerated wings after genetic ablation and Pi3K pathway inhibition alone or in combination with *Ask1* mutant backgrounds (genotypes indicated). Right: *sal*^*E/Pv*^*>rpr* ON, examples of wings with full regeneration (controls *sal*^*E/Pv*^*>rpr* or *ci>dp110*^*DN*^ alone) and anomalous regeneration (*sal*^*E/Pv*^*>rpr* with the genotypes indicated); *sal*^*E/Pv*^*>rpr* OFF wings: examples of control wings in the absence of *rpr*-ablation (maintained at 17°C). Error bars indicate standard error of sample proportion. ***P<0.001.

We first found that the active phosphorylated form of Akt (P-Akt) was increased in the living tissue and decreased or was absent in the dying zone (Fig 3A–3D). To test whether the Akt1 phosphorylated the endogenous Ask1 Ser83 *in vivo*, we specifically inhibited this kinase with a *UAS-Akt*^*RNAi*^ in a stripe of cells at the center of the disc, the *ptc* domain (*ptc>Akt*^*RNAi*^), and found that P-Ser83 was reduced (Fig 3E and 3F). In addition, ectopic activation of Akt1 (*UAS-myr-Akt1*.*S*), a constitutively activated membrane-anchored form of *Akt1* [50], resulted in an increase in the levels of P-Ser83 (Fig 3G and 3H).

We next studied the role of P-Akt on Ask1 in the context of regeneration. In the presence of cell death and blocking *Pi3K92E*, with the dominant negative form *dp110*^*DN*^ in the adjacent compartment, we observed a reduction in the accumulation of P-Akt and P-Ser83 induced after genetic ablation (*sal*^*E/Pv*^*>rpr hh> dp110*^*DN*^ in Fig 3I–3K). Moreover, in the overlapping zone of dying cells and *dp110*^*DN*^, the accumulation of P-Thr was unaffected (Fig 3L and S5 Fig). We also scored the effects on wing regeneration after blocking *Akt* and *Pi3K92E* kinases. Regeneration was impaired when the dominant negative form of *Pi3K92E* was expressed in the anterior compartment (*sal*^*E/Pv*^*>rpr ci>dp110*^*DN*^) as well as in the *Akt1*^*1*^ heterozygous background *(sal*^*E/Pv*^*>rpr Akt*^*1/+*^*)*, an allele that encodes a catalytically inactive protein [51] (Fig 3M). Moreover, regeneration was severely affected in double heterozygous flies containing *Akt1*^*1*^ and *Ask1*^*MB06487*^ or *Ask1*^*MI02915*^ alleles (Fig 3M). Neither the dominant negative form of *Pi3K92E* nor the allelic combination *Akt1*^*1*^ and *Ask1*^*MB06487*^ or *Ask1*^*MI02915*^ affected wing development in the absence of cell death (*sal*^*E/Pv*^*>*OFF wings in Fig 3M). These results further support the notion that Pi3K92E/Akt1 and Ask1 genetically interact and that their epistatic interaction is key to drive regeneration.

### ROS promote Ask1 activation

**Fig 4:**
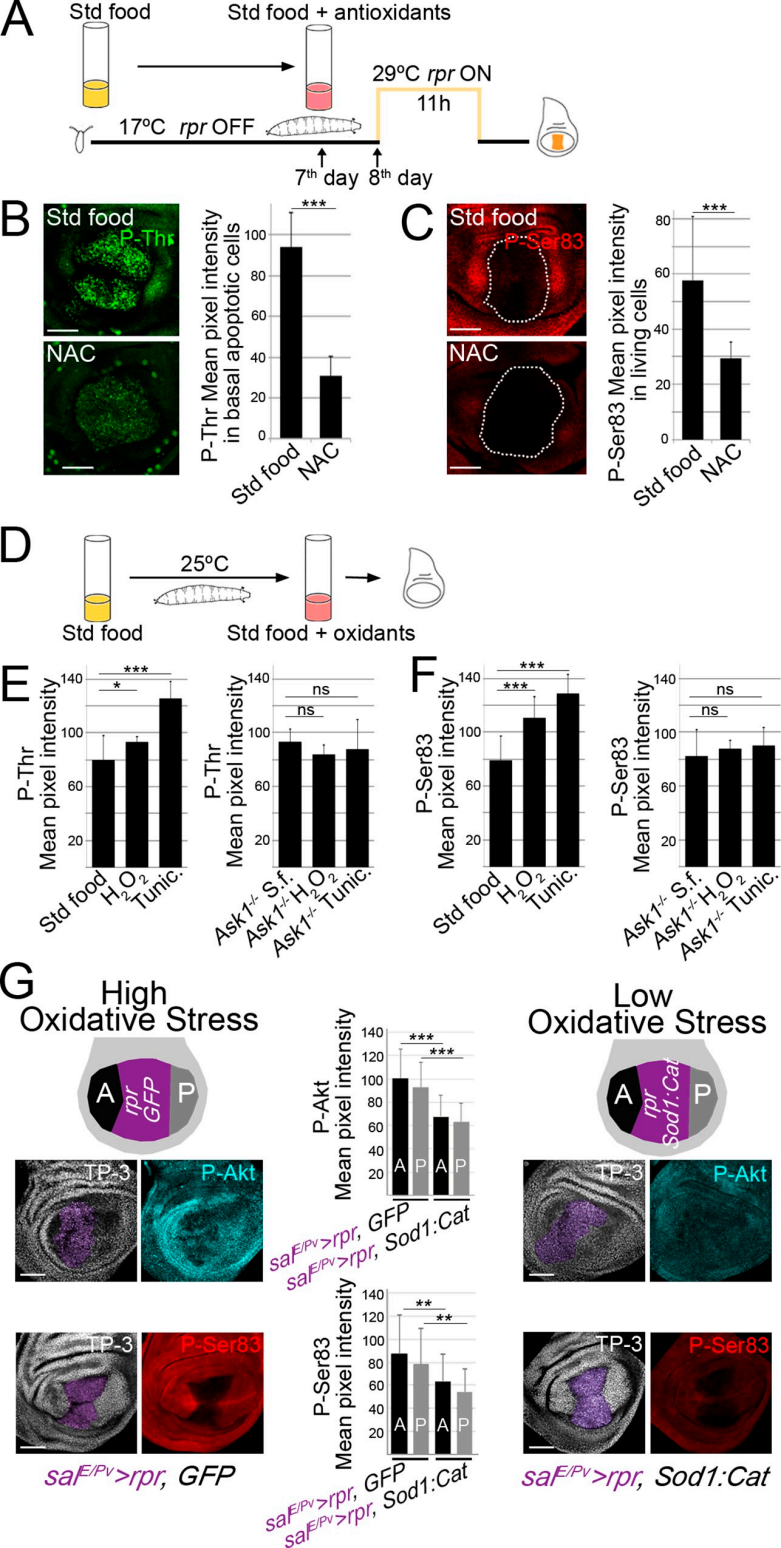
Ask1 is activated by ROS. (A) Design of experiments after feeding larvae with the NAC antioxidant. (B) Examples of Ask1 P-Thr after *sal*^*E/Pv*^*>rpr* induction in standard or NAC-supplemented food. Mean pixel intensities of P-Thr fluorescent labeling in the apoptotic zone of *sal*^*E/Pv*^*>rpr* discs from larvae fed with a standard or NAC-supplemented diet. (C) Examples of Ask1 P-Ser83 after *sal*^*E/Pv*^*>rpr* induction with standard or NAC-supplemented food. White dotted line indicates the *sal*^*E/Pv*^*>rpr* dying domain. Mean pixel intensities of Ask1 P-Ser83 fluorescent labeling in the neighboring cells near the apoptotic zone of *sal*^*E/Pv*^*>rpr* discs from larvae fed with a standard or NAC-supplemented diet. (D) Design of experiments after enhancing oxidative stress. (E) Left: P-Thr mean pixel intensity of wild-type wing discs from larvae fed with standard, H_2_O_2_- and tunicamycin-supplemented food. Right: P-Thr mean pixel intensity of *Ask1*^*MB06487*^ homozygous mutant discs from larvae fed with standard, H_2_O_2_- and tunicamycin-supplemented food. (F) Left: P-Ser83 mean pixel intensity of wild-type wing discs from larvae fed with standard, H_2_O_2_- and tunicamycin-supplemented food. Right: P-Ser83 mean pixel intensity of *Ask1*^*MB06487*^ homozygous mutant discs from larvae fed with standard, H_2_O_2_- and tunicamycin-supplemented food. (G) P-Akt (top) and P-Ser (bottom) mean pixel intensity from discs with *rpr* activation (*sal*^*E/Pv*^*>rpr*, *GFP;* high oxidative stress) and from discs with simultaneous *Sod1*:*Cat* and *rpr* activation (*sal*^*E/Pv*^*>rpr*, *Sod1*:*Cat;* low oxidative stress). Purple area shows where transgenes were activated. A and P are the zones where pixel intensity was measured. S.f.: standard food. *P<0.05. **P<0.01, ***P<0.001. Error bars indicate standard deviation. TP-3: TO-PRO-3 nuclear staining. Scale bars: 50μm.

Next, we modulated the ROS levels in order to determine if Ask1 can sense oxidative stress after damage. We fed *sal*^*E/Pv*^*>rpr* larvae with food supplemented with N-acetyl cysteine (NAC), a potent non-enzymatic scavenger that decreases ROS production, and examined Ask1 phosphorylation (Fig 4A). After induction of cell death, we found a significant decrease in both P-Thr and P-Ser83 in discs from NAC-fed larvae (Fig 4B and 4C; S3 Appendix). In addition, we fed larvae with H_2_O_2_-supplemented food and observed a significant increase in both P-Thr and P-Ser83 levels (Fig 4D–4F). This oxidative stress-induced increase was blocked in the hypomorphic *Ask1*^*MB06487*^ homozygous mutant (Fig 4E and 4F; S3 Appendix). It is known that in mammalian cells Ask1 can also be activated by endoplasmic reticulum (ER) stress [52]. Feeding larvae with tunicamycin, an inhibitor of N-glycosylation in the ER that induces ER stress, led to an increase in Ask1 activation, which was also blocked in *Ask1*^*MB06487*^ mutant discs (Fig 4E and 4F; S3 Appendix).

Furthermore, we examined whether Akt1 activation in the living cells is targeted non-autonomously by ROS produced by the dying cells. To examine this hypothesis, we enzymatically blocked ROS production using ectopic expression of the ROS scavengers *Superoxide dismutase 1* and *Catalase* (*Sod1*:*Cat*) in the *rpr* ablated region (*sal*^*E/Pv*^*>rpr*, *Sod1*:*Cat)* and monitored the pixel intensities of two adjacent zones in each disc, the anterior (A) and posterior (P) to the *sal*^*E/Pv*^ (Fig 4G). The activation of the *Sod1*:*Cat* transgene concomitantly with *sal*^*E/Pv*^*>rpr* did not inhibit apoptosis and P-Thr was detected in the dying cells (S6 Fig). However, in these conditions, we found that P-Akt leveis in neighboring cells were reduced in both A and P zones when *Sod1*:*Cat* was co-expressed with *rpr*. Accordingly, we found that the increase of P-Ser83 in neighboring cells was blocked when *Sod1*:*Cat* was co-expressed with *rpr* (Fig 4G; S3 Appendix). Together, these results demonstrate that the oxidative stress generated from the dying cells targets Akt1 and Ask1 in the neighboring undamaged tissue.

### Ask1 activates p38 and JNK in regeneration

**Fig 5:**
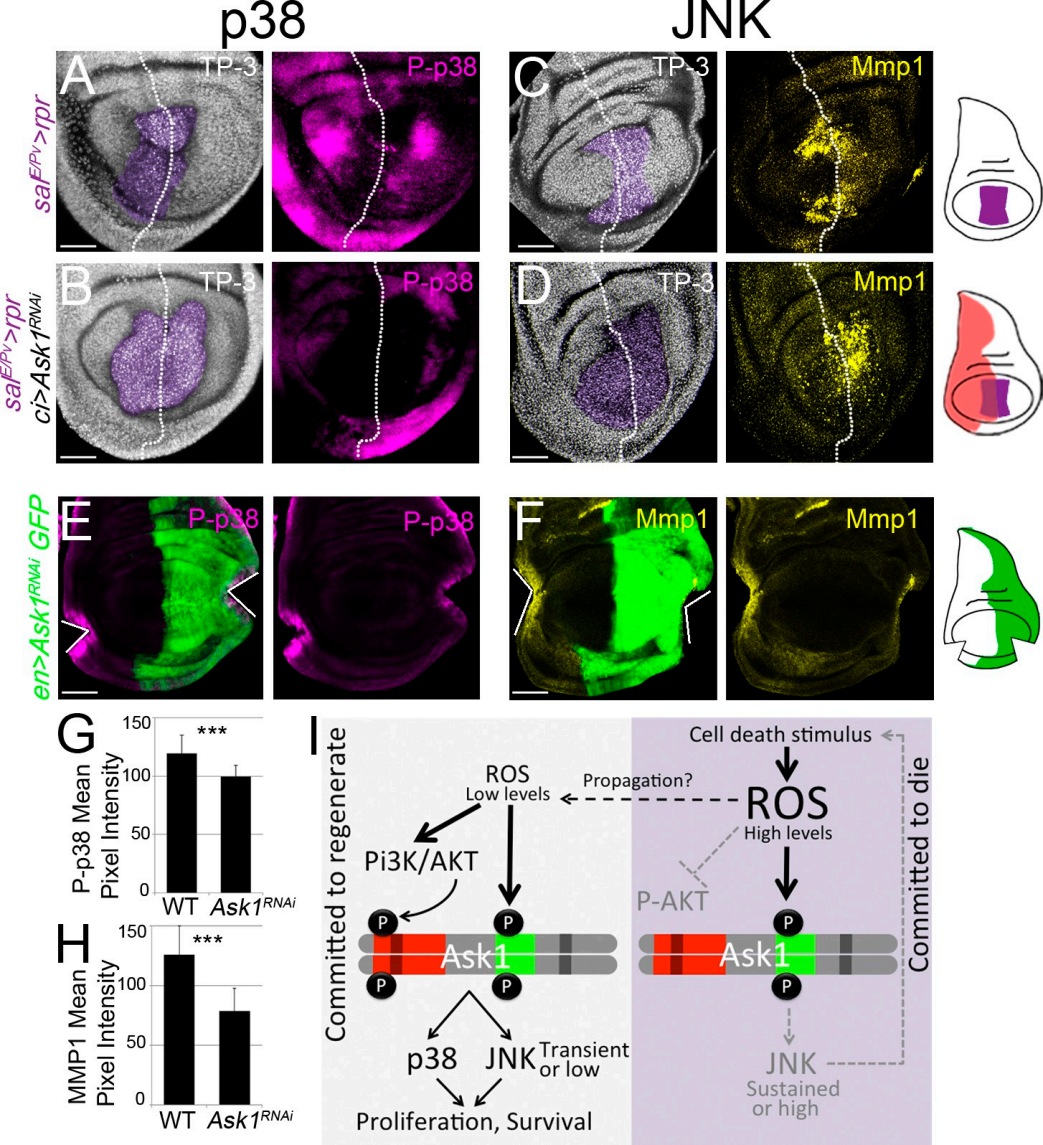
Ask1 promotes JNK and p38 signaling. (A) P-p38 after *sal*^*E/Pv*^*>rpr* induction (n = 14). (B) Inhibition of *Ask1* in the anterior compartment (*sal*^*E/Pv*^*>rpr ci>Ask1*^*RNAi*^) stained for P-p38 (n = 7). (C) Mmp1 after *sal*^*E/Pv*^*>rpr* induction (n = 16). (D) Inhibition of *Ask1* in the anterior compartment (*sal*^*E/Pv*^*>rpr ci>Ask1*^*RNAi*^) stained for Mmp1 (n = 14). TP-3: TO-PRO-3 nuclear staining. Purple: *sal*^*E/Pv*^*>rpr* ablated zone. White dotted line divides the discs into anterior (*Ask1* inhibition; to the left) and posterior (wild-type; to the right) compartments. (E-H) The *UAS-Ask1*^*RNAi*^ was activated in the posterior compartment together with *UAS-GFP* (green). Two injuries were inflicted with tungsten needles in Schneider’s medium, one in the anterior and one in the posterior compartment. Discs were stained for P-p38 and Mmp1. (E) For P-p38 staining, discs were immediately fixed after physical injury. (F) For Mmp1 staining, discs were first cut and cultured for 5 hours before fixation and processing. White lines indicate the wound site. Scale bar: 50μm. (G) Quantification of P-p38 activation at the wound site (n = 10 discs each genotype). (H) Quantification of Mmp1 at the wound site (n = 8 discs each genotype). Error bars indicate standard deviation. ***P<0.001. (I) Model proposed for the onset of regeneration in the columnar epithelium of the wing imaginal disc.

We have shown that the Ask1 P-Ser83 attenuating signal is essential for regeneration, and that this signal depends on ROS and Akt. We next hypothesized that this P-Ser83-mediated attenuation does not silence Ask1, but instead maintains the low levels of Ask1 activity necessary for regeneration. Therefore, we analyzed the expression of its known targets, the stress-activated MAP kinases JNK and p38 [48,53], both required for ROS-dependent regeneration [5,6]. We induced cell death in the *sal*^*E/Pv*^ domain and simultaneously blocked *Ask1* in the anterior compartment, and used the posterior as an internal control for the same disc (*ci>Ask1*^*RNAi*^ *sal*^*E/Pv*^*>rpr*). We found most phosphorylated p38 (P-p38), as an indicator of p38 activity, in the posterior compartment (Fig 5A and 5B). The effects of Ask1 on JNK activity were monitored by matrix metalloproteinase 1 (Mmp1) expression as it is one of the bona fide transcriptionally regulated read-outs of the JNK pathway [54]. After *sal*^*E/Pv*^*>rpr* cell death, Mmp1 was found in both compartments, but in *ci>Ask1*^*RNAi*^ *sal*^*E/Pv*^*>rpr* discs Mmp1 mostly accumulated in the posterior (Fig 5C and 5D). The effects of *Ask1* interference on JNK pathway were strengthened after staining with P-JNK antibody. P-JNK was found accumulated around the dead zone in *sal*^*E/Pv*^*>rpr* discs. However, when *Ask1* was knockdown in the anterior compartment (*ci>Ask1*^*RNAi*^ *sal*^*E/Pv*^*>rpr* discs), P-JNK was reduced in this compartment and accumulated in the posterior (S7A and S7B Fig). To confirm that Ask1 is responsible for JNK and p38 activation, we tested whether *Ask1* was required for their activation after physical damage. We made two incisions in *hh>GFP*,*Ask1*^*RNAi*^ discs, one in the *UAS-Ask1*^*RNAi*^ compartment and another into the control compartment. We found that P-p38 localized at the wound edges in the control compartment, whereas the levels of P-p38 were reduced at the wound edges of the *Ask1*^*RNAi*^ compartment (Fig 5E and 5G). Likewise, cut *Ask1*^*RNAi*^ discs resulted in less Mmp1 than in the control compartment (Fig 5F and 5H). In addition, we tested the *puckered* (*puc*) reporter of the JNK pathway, which normally accumulates in wounds, and found to drop in heterozygous *Ask1* discs after a physical injury (S7C and S7D Fig). Together, these results indicate that *Ask1* is necessary for the activation of p38 and JNK during regeneration.

## Discussion

We have shown here that Ask1 acts as a sensor of ROS after damage, and that synergizes with Pi3K/Akt1 to phosphorylate the Ask1 Ser83, a key phosphorylation event to initiate regeneration. In addition, we have demonstrated that the activation of the Pi3K/Akt1 and Ask1 in undamaged cells is originated non-autonomously by ROS produced by the damaged tissue.

An essential question to understand regeneration is how damaged cells communicate to the nearby living tissue to initiate regenerative growth. One of the most classical and determining studies on epithelial regeneration was the discovery that massive cell death caused by ionizing radiation in *Drosophila* larvae resulted in proliferation of the unaffected neighbors to compensate the lost [55]. Since then, compensatory proliferation, a cellular response linked to regeneration, has been considered as a result of signals released from apoptotic cells (reviewed in [56]). We propose here that the oxidative stress generated by damaged or apoptotic cells signals undamaged tissue to initiate the Ask1/Akt1 machinery that will culminate with repair and compensatory proliferation.

ROS acting in signaling after wounding has been subject of extensive research in various organisms and tissues [5,6,64–67,26,57–63]. Here, we have uncovered two scenarios of Ask1 activity: high activity in apoptotic cells, with high levels of P-Thr; and low activity in undamaged cells, where P-Ser83 prevails over P-Thr. This fits with the high levels of ROS and JNK in dying cells and the low levels of ROS, low JNK and P-p38 in living cells reported previously [5]. Together, our observations address the question of how JNK signaling selectively fosters apoptosis or proliferation, and how this signal is related to high or low ROS levels. The same applies to p38, which is only activated in unharmed cells when neighbors enter in apoptosis or are damaged [5,53].

Our study demonstrates that Ask1 operates in an Akt1-dependent manner in living cells and in an Akt1-independent manner in dying cells. It is conceivable that Akt1-mediated attenuation results in either low or transient levels of Ask1 activity necessary to stimulate P-p38 and low JNK levels for regeneration. In contrast, apoptotic cells induce high or sustained levels of JNK because high levels of ROS result in high Ask1 P-Thr in the absence of Akt1 attenuation.

Based on the results presented here and previous observations, we propose a model in which oxidative stress and the absence or presence of Pi3K/Akt1 survival signals are integrated in Ask1 to control the selective activity of JNK and p38, and in turn regenerative growth (Fig 5I). In this model, only dying cells produce high ROS levels and high JNK activity [5,7]. In addition, other mechanisms can exacerbate apoptosis through the lethal levels of JNK, and therefore increase ROS production. For example JNK, as well as p53, functions in a positive feedback after apoptosis as it transcriptionally activates the pro-apoptotic genes *hid* and *rpr*, thus amplifying apoptosis [31,68]. It is therefore likely that the high ROS that result in high Ask1 activation, inabilities the dying cells to respond to Pi3K/Akt1, preventing the Ask1 attenuation and resulting in deleterious JNK activity. We also have demonstrated that P-Akt1 is absent, or low, in dying cells, which supports a lack of Pi3K/Akt1-dependent Ask1 attenuated activity in dying cells. These cells will enter into a committed-to-die status with no return to survival. In contrast, the living neighbor cells, albeit adjacent to dying cells, show tolerable levels of ROS [5,7], and activate moderate levels of Ask1 through the Akt1-dependent phosphorylation of Ask1 P-Ser83. In these cells, Ask1 will turn on the activation of tolerable levels of JNK and p38, leading to a committed-to-regenerate status. Eventually, the JNK and p38 pathways will promote the transcription of the *unpaired* cytokines necessary for survival and regeneration [5,15,69–72]. In addition to cytokines, Wnt/Wg signaling pathway acts as a mitogenic signal involved in wing disc regeneration that responds to JNK [11,18,69,73] as well as the inhibition of the Hippo pathway, which results in increased Yki activity [74–76].

Several phosphatases are sensitive to ROS, and could be also integrated into the model presented here. This is the case of the JNK phosphatases, which are critical molecular targets of ROS. Oxidative inhibition of JNK phosphatases results in sustained JNK activation in order to promote caspase 3 cleavage and eventually cell death [77]. Therefore, the early burst of ROS in dying cells could activate lethal levels of JNK activity through inhibition of the *Drosophila* JNK phosphatase *puckered* concomitantly with the high levels of Ask1, and together boost the committed-to-die status. Additionally, the PTEN phosphatase, which regulates many cellular processes through direct antagonism of Pi3K signaling, is also ROS sensitive. Increasing ROS can enhance insulin signaling attributable to the oxidative inhibition of PTEN [78]. PTEN inactivation enhances the activation of AKT signaling, which in turn promotes the expression of cell survival genes [78]. Thus, PTEN could be involved in the living cells to release the Akt1 activity that is necessary to reach the tolerable levels of Ask1/JNK/p38. Therefore, the low oxidative stress that reaches the living cells can trigger not only Trx2 dissociation from Ask1, but also the PTEN-oxidation dependent Pi3K/Akt1 activity required for attenuated Ask1 necessary for survival and proliferation.

Our model concerns to an inherent response in the imaginal disc epithelium. However, other signals could enhance or stabilize the ROS-dependent regenerative response. For example, ROS produced after wounding are necessary for recruitment and activation of the *Drosophila* macrophage-like immune cells, which will secrete the TNF ortholog Eiger. Eiger activates JNK signaling in the epithelial cells to contribute to the regenerative response [6].

Because the moderate activity of Ask1 for regeneration is achieved in an Akt1-dependent manner, and because Akt1 functions downstream of the insulin signaling, it seems conceivable an implication of nutrition in imaginal disc regeneration. The production and secretion of insulin and AKH, the fly analog of mammalian glucagon, vary depending of the food content. Thus, a challenge for the future will be to analyze how these hormones can modulate the regenerative response to damage. For example, the *Drosophila* TNF Eiger is produced by the fat body when larvae are exposed to low protein diet [79]. As Eiger activates JNK non-autonomously in many tissues, it could have a central role in imaginal disc regeneration, depending on food availability. The relation between nutrition and metabolism, as well as their involvement in regeneration deserves further analysis in order to achieve an integrative view of regeneration.

## Materials and methods

### *Drosophila* strains

The *Drosophila melanogaster* strains, *sal*^*E/Pv*^*-LHG* and *lexO-rpr*, are previously described [5]; *lexO-GFP* (gift from K. Basler). *UAS-H2B-RFP* (from J. Knoblich), *Akt1*^*1*^(gift from H. Stocker), *sal-Gal4*, *sal*^*E/Pv*^*-Gal4* (gift from J.F. de Celis), *ci-Gal4* (from R. Holmgren) and *en-Gal4* (gift from G. Morata). The following strains were provided by the Bloomington Drosophila Stock Center: *tubGal80*^*TS*^ (RRID:BDSC_7017), *ptc-Gal4* (*RRID*:*BDSC_2017*), *Ask1*^*MB06487*^ (RRID:BDSC_26048) *Ask1*^*MI02915*^ (RRID:BDSC_36163), *UAS-GFP* (RRID:BDSC_4776), *UAS-dp110*^*DN*^ (RRID:BDSC_25918), *Df(3R)BSC636* (RRID:BDSC_25726), *UAS-Ask1*^*RNAi*^ (RRID:BDSC_35331), *UAS-Cat*.*A* (RRID:BDSC_24621), *UAS-Sod*.*A (Sod1)* (RRID:BDSC_24754), *UAS-rpr* on the X chromosome (RRID:BDSC_5823), *UAS-rpr* on the third chromosome(RRID:BDSC_50791). These strains are described in FlyBase: *hh-Gal4*, *ap-Gal4*, *UAS-hid*. The *UAS-Akt*^*RNAi*^ (2902) strain was obtained from the Vienna Drosophila Resource Center (VDRC). We used the *UAS-myr-Akt1*.*S* [50]. Canton S and *w*^*118*^ were used as controls. A full list of genotypes is provided at the end of this section.

### Genetic ablation and dual Gal4/LexA transactivation system

Cell death was genetically induced as previously described [5]. We used the *sal*^*E/Pv*^*-Gal4* as a driver, which consists of the *spalt* wing enhancer with expression confined to the wing to score adult wing parameters. The UAS lines used to promote cell death were *UAS-rpr* or *UAS-hid*, two pro-apoptotic genes, controlled by the thermo-sensitive Gal4 repressor *tubGal80*^*TS*^. We also used the *sal*^*E/Pv*^*-LHG* and *LexO-rpr* strains [5] for genetic ablation, utilizing the same design as for Gal4/UAS.

Embryos were kept at 17°C until the 8th day/192 hours after egg laying to prevent *rpr* expression. They were subsequently moved to 29°C for 11 hours and then back to 17°C to allow tissue to regenerate. Two types of controls were always treated in parallel; individuals without *rpr* expression (*UAS-GFP*, moved to 29°C for 11 hours) and individuals kept continuously at 17°C to avoid any transgene activation.

In most of the experiments shown here, we induced cell death for 11h. However the induction of cell death for testing the effects of signals emerging from the dying zone was longer. This is the case of the P-Akt experiment in Fig 3B and 3C and for the genetic scavenging of ROS (*Sod1*:*Cat*) (Fig 4G and S6 Fig), in which *sal>rpr* was activated for 24h.

In dual transactivation experiments, we used *sal*^*E/Pv*^*-LHG LexO-rpr* to ablate the *sal*^*E/Pv*^ domain (abridged as *sal*^*E/Pv*^*>rpr*), whereas Gal4 was used to express different transgenes under the control of Gal4 drivers (*ci-Gal4* for anterior compartment; *ap-Gal4* for dorsal compartment and *hh-Gal4* for posterior compartment).

### Test for regenerated adult wings and statistics

To test the capacity to regenerate in different genetic backgrounds, we used adult wings emerged from *sal*^*E/Pv*^*>rpr* individuals in which patterning and size defects can be scored easily. Flies were fixed in glycerol:ethanol (1:2) for 24 hours. Wings were dissected in water and then washed with ethanol. Subsequently, they were mounted in lactic acid:ethanol (6:5) and analyzed and imaged under a microscope.

The percentage of regenerated wings refers to fully regenerated (for genetic ablation genotypes) or normally developed wings (for testing transgenes) and was calculated according to the number of wings with a complete set of veins and interveins, as markers of normal patterning. For each sample, we scored the percentage of individuals belonging to the “regenerated wings” class. We calculated the standard error of the sample proportion based on a binomial distribution (regenerated complete wing or not) SE = *√*p (1-p)/n, where p is the proportion of successes in the population.

### Computational characterization of the fly Ser83 domain

Serine/threonine putative phosphorylation sites were determined by scanning the orthologous human/fly sequences with the RxRxx[ST] domain motif for the Akt kinase (purple block on S2 Fig) and the degenerated pattern xxRxx[ST] (green blocks on S2 Fig). Strikingly the human Ser83 site was not found on *Drosophila melanogaster*; which was confirmed by using GeneWise (version wise2.4.1) [80] to map the M3K5_HUMAN protein sequence (Q99683), downloaded from UniProt [81], over a window of 10kbp centered at the Ask1 transcription start site; this sequence segment was retrieved from FlyBase FB2017_06 genome version ([82], chr3R:19,875,000–19,885,000[+]). The GeneWise output is provided as S1 Appendix, the software tool was not able to find a match on the fly genome for the initial 126 residues from the human amino term. In order to assess the relevance of the fly Ser83 two homology-based approaches were implemented. First, a search on PFAM database [83] returned the domain of unknown function DUF4071 (PF13281). The first amino acid positions for this domain are highly conserved across a set of diverse homologs, including fly Ask1-PC and human MAP3K5 but also paralogs sharing the domain. The consensus sequence for DUF4071 has been included on the right alignment block from S2 Fig for illustrative purpose. The second approach was considering a precomputed set of homolog sequences downloaded from EggNOG database (version 4.5) [84]. A cluster of homologous sequences already included fly Ask1 (KOG4279), providing a total of 338 protein sequences for 164 species; a total of 49 sequences were selected from that set, fly Ask1-PC isoform among them, that were also reliably annotated as orthologs of human MAP3K5. Fly Ask1-PB isoform protein sequence was retrieved from FlyBase and appended to the selected MAP3K5 orthologs. Then, the 50 protein sequences were aligned by MAFFT (version 7.271) [85] using the following parameters: maxiterate = 1000, localpair, op = 10. The complete alignment is available from S2 Appendix, from which two conserved blocks centered on the human and fly Ser83 domains were trimmed and processed with TeXshade [86] to produce the S2 Fig. Note that on S2 Fig the domain highlighted in cyan, which includes the fly equivalent column highlighted in red, is highly conserved in a wide range of taxa, from sponges to humans. Other domains are conserved only within chordata or even tetrapoda species. Such conservation signature makes *Drosophila* Ser83 a good candidate for functional and mutational studies.

### Generation of *UAS-Ask1*^*WT*^ and *UAS-Ask1*^*S83A*^ flies

pUASt-attb_Ask1^WT^ was constructed by cutting Ask1 cDNA EcoRI /BamHI from DGRC clone FI02066 and cloning it into pUASt-attb.

pUASt-attb_Ask1^S83A^. Two serines in the 75 and 83 residues in the DUF4071 domain showed putative non-canonical AKT phosphorylation sites (ILTQQRPLSYHYGVRESF). Both residues are spatially exposed similarly to human Ser83 of ASK1, which makes them accessible to kinases. pUASt-attb_Ask1 S83A was constructed by mutating serine 83 to alanine by PCR using oligos Ask1Mut-Fwd and Ask1S83A-Rev for partial PCR1 and Ask1S83A-Fwd and Ask1Mut-Rev for partial PCR2. Complete PCR was performed using the two partial PCRs as templates with oligos Ask1Mut-Fwd and Ask1Mut-Rev. The complete PCR was then cut with EcoRI /PflMI and cloned into FI02066-cut EcoRI/PflMI. The mutation introduced a new StuI site that was used to check the mutated clones. Mutated Ask1S83A cDNA was then cut from EcoRI/BamHI and cloned into pUASt-attb. Both clones were injected by standard procedures in line zh-86Fb-attP and transgenic lines were selected.

Ask1Mut-Fwd: AAT ACA AGA AGA GAA CTC TGA ATA CGG AAT

Ask1Mut-rev: CGG CGG TGT GGT TTT GTG CAC AAA CCG ATC

Ask1S83A-Fwd: CGT TAG GGA GGC CTT CGG GAT GAA GGA GA

Ask1S83A-Rev: CGG CGG TGT GGT TTT GTG CAC AAA CCG ATC

### Immunochemistry

Immunostaining was performed using standard protocols. The primary antibodies used in this study were the polyclonal Ask1 P-Ser83 (Santa Cruz Biotechnology sc-101633 1:100, which recognizes the conserved region surrounding human P-Ser83) and Ask1 P-Thr (Santa Cruz Biotechnology sc-109911 1:100, which labels the preserved region nearby mouse P-Thr845). We selected the anti-Ask1 P-Ser83 because of the following in vivo analysis: (1) its localization dropped under a *UAS-Ask1*^*RNAi*^ (S3C Fig); (2) Its localization increased after ectopic activation of *Ask1*^*WT*^ (Fig 2G); (3) Mutation in the *Drosophila* Ser83 residue (*UAS-Ask1*^*S83A*^), inhibited its localization (Fig 2H); (4) Akt activation or repression, resulted in increased or decreased localization of P-Ser83 (Fig 3E–3H). Other antibodies used were: Ptc (DSHB, 1:100), P-p38 (Cell Signalling 1:50), Mmp1 (cocktail of three antibodies: DSHB 3A6B4, 5H7B11, 3B8D12 1:100), P-Akt (S473 Cell Signalling 1:100) and P-Histone-H3 (Millipore, 1:1000).

The anti-P-JNK used was the Anti-ACTIVE® JNK pAb, Rabbit, (V7931, Promega, 1:100). The images were taken using the Thermal LUT from the 3D Surface Plot of FIJI, with maximum values of 50% and minimum 24% to minimize the background. Calculation of pixel intensities was obtained from raw images.

Fluorescently labeled secondary antibodies were from ThermoFisher Scientific. Discs were mounted in SlowFade or ProLong (ThermoFisher Scientific) supplemented with 1 μM TO-PRO-3 (TP-3) or YO-PRO-1 (YP1) (Life Technologies) to label nuclei. For apoptotic cell detection, we used the TUNEL assay. We employed the fluorescently labeled Alexa Fluor® 647-aha-dUTP (ThermoFisher Scientific), incorporated using terminal deoxynucleotidyl transferase (Roche).

The number of mitotic cells per mm^2^ (Fig 1C) was calculated after counting the number of P-Histone-H3 (P-H3) positive cells in the anterior compartment of the wing pouch and hinge, excluding the notum, for all the genotypes shown. Note that two genotypes in Fig 1C show the anterior compartment labeled with the transgene *ci-Gal4 UAS-GFP* (*ci>GFP*), whereas in the two *ci>Ask1*^*RNAi*^ experiments the anterior compartment was labeled with anti-Ci. In these experiments shown in Fig 1C, the induction of cell death or activation of transgenes was done for 16h at 29°C. The number of mitosis after analyzing the stacks of confocal images was calculated using Fiji software.

### Imaginal disc culture and physical injury

Wing discs were dissected from third instar larvae in Schneider’s insect medium (Sigma-Aldrich), and a small fragment was removed with tungsten needles. To visualize Mmp1 staining, the discs were cultured in Schneider’s insect medium supplemented with 2% heat-activated fetal calf serum, 2.5% fly extract and 5 μg/ml insulin, for 5 hours at 25°C. For P-p38, discs were injured in Schneider’s insect medium and immediately fixed and stained. *Ex vivo* images were taken using a Leica SPE confocal microscope and processed with Fiji software.

*Ex vivo* discs were also monitored live after a cut, using *puc-Gal4 UAS-GFP* as a JNK reporter, using the same medium. For nuclei visualization in *ex vivo* culture, we used NucRed Live647 (Life Technologies; 1 drop per slide) added 20 minutes before imaging.

### ROS scavenging

To prevent ROS production in *sal*^*E/Pv*^*>rpr* discs we pursued two different protocols. First, we inhibited ROS chemically (Fig 4A–4F). To do this, standard fly food was supplemented with the antioxidant N-acetyl cysteine (NAC 100 μg/ml; Sigma-Aldrich). NAC treatment was dispensed on the 7^th^ day of development at 17°C. On the 8^th^ day, experimental larvae were moved to 29°C for 11 hours to promote cell death, whereas controls were transferred to a vial with standard food and moved to 29°C for the same time period. Afterward, the larvae were move back to 17°C to allow tissue recovery. Second, we decreased ROS production genetically by the ectopic expression of the Sod1 and Catalase enzymes using a recombinant fly *UAS-Sod1*:*UAS-Cat* (*Sod1*:*Cat*) in the *sal*^*E/Pv*^*>rpr* domain (Fig 4G and S6 Fig). For those experiments, *Sod1*:*Cat* and *rpr* were activated for 24 hours at 29ºC.

### Oxidative stress induction

Third instar larvae were transferred to vials with 5mL of special medium containing 1.3% UltraPure^TM^ LMP agarose (Invitrogen), 5% sucrose (Fluka) and the desired concentration of 0.1% H_2_O_2_ (Merck) or 1ng/μl tunicamycin (Sigma-Aldrich). To avoid loss of oxidative capacity, these substances were added to the media at a temperature below 45°C. The larvae were fed for 2 hours prior to dissection and fixation of the discs. Controls without H_2_O_2_ or tunicamycin were always handled in parallel.

### Quantitative real-time PCR analysis

Control (*hh>RFP*) and experimental (*hh>Ask1*^*RNAi*^) conditions were induced for 16h at 29°C (192h after egg laying) using the *Gal4/tub-Gal80*^*TS*^ system. Wing imaginal discs (n = 40) were dissected after induction in three biological replicates for each condition. RNA was extracted with the Zymo Research ZR RNA MicroPerp (R1060/R1061) and RNA Clean and Concentrator (R1015/R1016) kits following standard protocols. RT-PCR was performed using SYBR green Master Mix (Roche). Specific primers for *Ask1* and *Rps18* are detailed in S1B Fig. ΔΔCt method was used to normalize the data. *Ask1*^*RNAi*^ and control samples were normalized against the housekeeping gene *Rps18*. Average standard error of the mean (SEM) of the three biological replicates was computed for each one based on three technical replicates by ΔΔCt method.

### Genotypes

**Fig 1**.

(A) *+/+* → *wUAS-rpr/+; sal*^*E/Pv*^*-Gal4/+; tubGal80*^*TS*^*/+*

*Ask1*^*MI02915*^*/+* → *wUAS-rpr/+; sal*^*E/Pv*^*-Gal4/+; tubGal80*^*TS*^*/Ask1*^*MI02915*^

*Ask1*^*MB06487*^*/+* → *wUAS-rpr/+; sal*^*E/Pv*^*-Gal4/+; tubGal80*^*TS*^*/Ask1*^*MB06487*^

(B) *sal*^*E/Pv*^*>rpr* → *w; ci-Gal4/LexO-rpr; sal*^*E/Pv*^*-LHG*:*tubGal80*^*TS*^*/UAS-GFP*

*ci>Ask1*^*RNAi*^ → *w; ci-Gal4/LexO-GFP; sal*^*E/Pv*^*-LHG*:*tubGal80*^*TS*^*/UAS-Ask1*^*RNAi*^

*sal*^*E/Pv*^*>rpr ci> Ask1*^*RNAi*^ → *w; ci-Gal4/LexO-rpr; sal*^*E/Pv*^*-LHG*:*tubGal80*^*TS*^*/UAS- Ask1*^*RNAi*^

*ap> Ask1*^*RNAi*^ → *w; ap-Gal4/LexO-GFP; sal*^*E/Pv*^*-LHG*:*tubGal80*^*TS*^*/UAS- Ask1*^*RNAi*^

*sal*^*E/Pv*^*>rpr ap> Ask1*^*RNAi*^ → *w; ap-Gal4/LexO-rpr; sal*^*E/Pv*^*-LHG*:*tubGal80*^*TS*^*/UAS- Ask1*^*RNAi*^

(C) *sal>GFP ci>RFP* → *w; ci-Gal4/LexO-GFP; sal*^*E/Pv*^*-LHG*:*tubGal80*^*TS*^*/UAS-RFP*

*sal>GFP ci>Ask1*^*RNAi*^ → *w; ci-Gal4/LexO-GFP; sal*^*E/Pv*^*-LHG*:*tubGal80*^*TS*^*/UAS-Ask1*^*RNAi*^

*sal*^*E/Pv*^*>rpr ci>GFP* → *w; ci-Gal4/LexO-rpr; sal*^*E/Pv*^*-LHG*:*tubGal80*^*TS*^*/UAS-GFP*

*sal*^*E/Pv*^*>rpr ci> Ask1*^*RNAi*^ → *w; ci-Gal4/LexO-rpr; sal*^*E/Pv*^*-LHG*:*tubGal80*^*TS*^*/UAS- Ask1*^*RNAi*^

**Fig 2**.

(B,C) WT → Canton S

(D) *sal*^*E/Pv*^*>rpr* → *wUAS-rpr/+; sal*^*E/Pv*^*-Gal4/+; tubGal80*^*TS*^*/+*

(E,F) *sal*^*E/Pv*^*>rpr ci> Ask1*^*RNAi*^ → *w; ci-Gal4/LexO-rpr; sal*^*E/Pv*^*-LHG*:*tubGal80*^*TS*^*/UAS- Ask1*^*RNAi*^

(G) *hh>Ask1*^*WT*^, *GFP* → *w; UAS-GFP/+; hh-Gal4/UAS- Ask1*^*WT*^

(H) *hh>Ask1*^*S83A*^, *GFP* → *w; UAS-GFP/+; hh-Gal4/UAS- Ask1* ^*S83A*^

(I) *sal>GFP ci>Ask1*^*WT*^ → *w; ci-Gal4/LexO-GFP; sal*^*E/Pv*^*-LHG*:*tubGal80*^*TS*^*/UAS- Ask1*^*WT*^

*sal>GFP ci>Ask1* ^*S83A*^ → *w; ci-Gal4/LexO-GFP; sal*^*E/Pv*^*-LHG*:*tubGal80*^*TS*^*/UAS- Ask1* ^*S83A*^

*sal*^*E/Pv*^*>rpr* → *w; ci-Gal4/LexO-rpr; sal*^*E/Pv*^*-LHG*:*tubGal80*^*TS*^*/UAS-GFP*

*sal*^*E/Pv*^*>rpr ci> Ask1*^*WT*^ → *w; ci-Gal4/LexO-rpr; sal*^*E/Pv*^*-LHG*:*tubGal80*^*TS*^*/UAS- Ask1*^*WT*^

*sal*^*E/Pv*^*>rpr ci> Ask1* ^*S83A*^ → *w; ci-Gal4/LexO-rpr; sal*^*E/Pv*^*-LHG*:*tubGal80*^*TS*^*/UAS- Ask1*^*S83A*^

**Fig 3**.

A) Canton S

(B) *ptc>rpr* → *wUAS-rpr/+; ptc-Gal4*: *tubGal80*^*TS*^ */+*

(C) *sal>rpr* → *wUAS-rpr/+; sal-Gal4/+; tubGal80*^*TS*^*/+*

(D,E) *ptc>Akt*^*RNAi*^ → *w; ptc-Gal4*:*tubGal80*^*TS*^*/+;UAS-Akt*^*RNAi*^*/+*

(F,G) *ptc>myrAkt* → *w; ptc-Gal4*:*tubGal80*^*TS*^*/+; UAS-myrAkt/+*

(H-K) *sal*^*E/Pv*^*>rpr hh>dp110*^*DN*^ → *w; LexO-rpr /UAS-dp110*^*DN*^*; sal*^*E/Pv*^*-LHG*:*tubGal80*^*TS*^*/hh-Gal4*

(L) *ci>dp110*^*DN*^ → *w; ci-Gal4/UAS-dp110*^*DN*^*; sal*^*E/Pv*^*-LHG*:*tubGal80*^*TS*^*/+*

*+/+* → *w; LexO-rpr/ +; sal*^*E/Pv*^*-LHG*:*tubGal80*^*TS*^*/+* (control for LexO-rpr in the second chromosome) and *wUASrpr/+; sal*^*E/Pv*^*-Gal4; tubGal80*^*TS*^ (control for mutant backgrounds)

*sal*^*E/Pv*^*>rpr ci>dp110*^*DN*^ → *w; ci-Gal4/UAS-dp110*^*DN*^*; sal*^*E/Pv*^*-LHG*:*tubGal80*^*TS*^*/ LexO-rpr*

*sal*^*E/Pv*^*>rpr Akt*^*1/+*^ → *wUAS-rpr/+; sal*^*E/Pv*^*-Gal4; tubGal80*^*TS*^*; Akt*^*1*^*/+*

*sal*^*E/Pv*^*>rpr Ask1*^*MB06487/+*^ *Akt*^*1/+*^ → *wUAS-rpr/+; sal*^*E/Pv*^*-Gal4; tubGal80*^*TS*^*; Akt*^*1*^*/Ask1*^*MB06487*^

*sal*^*E/Pv*^*>rpr Ask1*^*MI02915/+*^ *Akt*^*1/+*^ → *wUAS-rpr/+; sal*^*E/Pv*^*-Gal4; tubGal80*^*TS*^*; Akt*^*1*^*/Ask1*^*MI02915*^

**Fig 4**.

(B,C) Std food and NAC → *wUAS-rpr/+; sal*^*E/Pv*^*-Gal4/+; tubGal80*^*TS*^*/+*

(E,F) CTRL → *w*^*118*^*; +; +*

*Ask1*^*-/-*^ → *w*^*118*^*; +; Ask1*^*MB06487*^*/Ask1*^*MB06487*^

(G) *sal>rpr*, *GFP* → *w; sal*^*E/Pv*^*-Gal4/UAS-GFP; tubGal80*^*TS*^*/UAS-rpr*

*sal>rpr*, *Sod1*:*Cat* → *w; sal*^*E/Pv*^*-Gal4/UAS-Sod1*:*UAS-Cat; tubGal80*^*TS*^*/UAS-rpr*

**Fig 5**.

(A,C) *sal*^*E/Pv*^*>rpr* → *w; LexO-rpr/ +; sal*^*E/Pv*^*-LHG*:*tubGal80*^*TS*^*/+*

(B,D) *sal*^*E/Pv*^*>rpr ci>Ask1*^*RNAi*^ → *w; ci-Gal4/LexO-rpr; sal*^*E/Pv*^*-LHG*:*tubGal80*^*TS*^*/UAS-Ask1*^*RNAi*^

(E-H) *en>Ask1*^*RNAi*^, *GFP* → *w; en-Gal4/UAS-GFP; UAS-Ask1*^*RNAi*^ */+*

**S1 Fig**.

(A) *sal*^*E/Pv*^*>rpr Ask1*^*MI02915/+*^ → *wUAS-rpr/+; sal*^*E/Pv*^*-Gal4/+; tubGal80*^*TS*^*/Ask1*^*MI02915*^

*sal*^*E/Pv*^*>rpr Ask1*^*MB06487/+*^ → *wUAS-rpr/+; sal*^*E/Pv*^*-Gal4/+; tubGal80*^*TS*^*/Ask1*^*MB06487*^

*sal*^*E/Pv*^*>rpr ci> Ask1*^*RNAi*^ → *w; ci-Gal4/LexO-rpr; sal*^*E/Pv*^*-LHG*:*tubGal80*^*TS*^*/UAS- Ask1*^*RNAi*^

(B) Rt-q-PCR

Controls: *w; tubGal80*^*ts*^*/+; hh-Gal4/UAS-RFP*

*UAS-Ask1*^*RNAi*^: *w; tubGal80*^*ts*^*/+; hh-Gal4/UAS-Ask1*^*RNAi*^

**S3 Fig**.

(A) *w*^*118*^*; +; +*

(B) *Ask1*^*+*^*/Ask1*^*+*^ → *w*^*118*^*; +; +*

*Ask1*^*MB06487*^*/Ask1*^*MB0647*^ → *w*^*118*^*; +; Ask1*^*MB06487*^*/Ask1*^*MB06487*^

*Ask1*^*MB06487*^*/Def(3R)BSC636* → *w*^*118*^*; +; Ask1*^*MB06487*^*/Def(3R)BSC636*

(C) *hh>Ask1*^*RNAi*^,*GFP* → *w; UAS-GFP/+; hh-Gal4/UAS- Ask1*^*RNAi*^

**S4 Fig**.

(A) wt and *ptc>rpr* → *wUAS-rpr/+; ptc-Gal4*: *tubGal80*^*TS*^ */+*

(B) *sal*^*E/Pv*^*>hid* → *w; sal*^*E/Pv*^*-Gal4/+; tubGal80*^*TS*^*/UAS-hi*

(C) wt and physically injured → Canton S

**S5 Fig**.

(A-D) *sal*^*E/Pv*^*>rpr ci>dp110*^*DN*^ → *w; ci-Gal4/UAS-dp110*^*DN*^*; sal*^*E/Pv*^*-LHG*: *tubGal80*^*TS*^*/ LexO-rpr*

**S6 Fig**.

w; sal^E/Pv^-Gal4/UAS-Sod1:UAS-Cat; tubGal80^TS^/UAS-rpr

**S7 Fig**.

(A) wt→Canton S

*sal*^*E/Pv*^*>rpr* → *w; LexO-rpr/+; sal*^*E/Pv*^*-LHG*: *tubGal80*^*ts*^

*sal*^*E/Pv*^*>rpr*, *ci>Ask1*^*RNAi*^→ *w; ci-Gal4/LexO-rpr; Sal*^*E/Pv*^*-LHG*: *tubGal80*^*ts*^*/UAS-Ask1*^*RNAi*^

(C) *w; UAS-GFP/+; puc-Gal4/+*

(D) *w; UAS-GFP/+; puc-Gal4/Ask1*^*MB06487*^

## Supporting information

S1 Fig Supporting information for Fig 1. (A) Spectrum of wings with anomalous regeneration, ranging from complete regeneration (*sal*^*E/Pv*^*>rpr* in wild type, top) to a severe effect found after *Ask1*^*RNAi*^ (*sal*^*E/Pv*^*>rpr* in mutant conditions, bottom). It includes a range of defects found after inducing cell death in Ask1 heterozygous background and after *UAS-Ask1*^*RNAi*^. Arrowheads indicate some defects. (B) Real time quantitative PCR of the *UAS-Ask1*^*RNAi*^. The table indicates the primers used. The RT-qPCR analysis is shown for *Ask1* expression in wing discs of controls (*hh>RFP*) and *Ask1*^*RNAi*^ (*hh>UAS-Ask1*^*RNAi*^). qPCR results are presented as relative quantity of mRNA between control and *Ask1*^*RNAi*^. Error bars represent the standard error of the mean from three biological replicates (n = 40 discs, each replicate).
(TIF)

S2 Fig Alignment of Ask1 *D*. *melanogaster* orthologs focusing on human and fly Ser83 residues. Two blocks of conserved columns pinpointing the aforementioned residues were selected from the whole-length alignment of 50 MAP3K5/Ask1 protein orthologs provided on the S2 Appendix. From top to bottom: sequence logos displaying information content based on amino acid frequencies; an estimate of hydrophobicity of the amino acid residues; the aligned sequences split into chordate and invertebrate sequences, above or below the two *Drosophila melanogaster* Ask1 isoforms respectively; finally, the similarity is depicted as a color gradient ranging from low (blue) to high (red). Bottom reference coordinates for the aligned residues are based on human reference sequence, which is listed on the first row. Serines, threonines, and arginines were labeled in red, green and blue when appearing outside the domain blocks, but shown in white and boldface within those blocks. Numbers on the left side of the alignment blocks correspond to the relative position of the first amino acid within those blocks for each species sequences. Left alignment block contains a domain in purple that matches the consensus RxRxx[ST] pattern, although conservation is limited to birds and mammals (dark versus light shades of violet). On the right, the domain highlighted in cyan is conserved all across the diverse taxa, from human (top) to sponges (bottom), and it contains a serine (red background) that corresponds to the fly Ser83. On both panels, another domain matching a degenerated xxRxx[ST] pattern that is only conserved within vertebrate sequences is shown as a green block. Right alignment also includes at the bottom the consensus sequence of a PFAM domain of unknown function (DUF4071) that starts two residues before the Ser83 conserved block; this block within the domain shows high conservation across the different protein families considered when building the corresponding model.
(TIF)

S3 Fig Supporting information for Fig 2. (A) Mitotic (P-H3 positive) and Ask1 P-Thr. Lower right images: Zoom of mitotic cells from the white square. TP-3: TO-PRO-3 nuclear staining. (B) The basal levels of P-Thr in wild-type (WT) discs (mitotic and interphase cells) decrease in the *Ask1*^*MB06487*^ homozygous mutant background and are strongly reduced in the *Ask1*^*MB06487*^ mutant background over the deficiency *Def(3R)50636*. Quantification of mean pixel intensity for P-Thr in mitotic and for P-Thr in non-mitotic (basal levels) cells are shown in separate graphics. P-Thr mean Pixel intensity in mitotic cells of the wild-type discs (+/+), 113.92±20.60, S.D. (n = 10); of the *Ask1*^*MB06487*^*/Ask1*^*MB06487*^ discs, 104.43±9.54, S.D. (n = 10); and of the *Ask1*^*MB06487*^*/Def(3R)BSC636* 88.37±13.03, S.D. (n = 10). Mean pixel intensity of the basal levels in interphase cells of the wild-type, 50.44±6.27, S.D. (n = 20); of the *Ask1*^*MB06487*^*/Ask1*^*MB06487*^, 41.65±4.80, S.D. (n = 10); and of the *Ask1*^*MB06487*^*/Def(3R)BSC636*, 27.22±5.73, S.D. (n = 18). (C) Inhibition of the endogenous Ask1 P-Ser83 levels after expressing the *UAS-Ask1*^*RNAi*^ in the posterior compartment, using the *hh-Gal4* driver. Scale bars: 5μm.
(TIF)

S4 Fig Supporting information for Fig 2. (A) Left: wild-type disc stained with P-Ser83. Right: disc in which apoptosis has been induced with the driver *ptc-Gal4 UAS-rpr* (*ptc>rpr*) and stained with P-Ser83 (n = 5). (B) S*al*^*E/Pv*^*>hid* discs stained with P-Thr (n = 12) and P-Ser83 (n = 5). YP-1: YO-PRO-1 nuclear staining. Purple: *sal*^*E/Pv*^*>rpr* ablated zone. (C) Staining of Ask1 P-Ser83 after physical injury, (n = 6). Square: magnified image. Scale bars: 50μm.
(TIF)

S5 Fig Supporting information for Fig 3. (A) Design of the experiments in B, C and D with *sal*^*E/Pv*^*>rpr* cell death (purple) and blocking Pi3K (*ci>dp110*^*DN*^) in the anterior compartment (black). (B-D) Staining of P-Akt (B), Ask1 P-Ser83 (C) and Ask1 P-Thr (D). White-dotted line indicates anterior-posterior boundary. TP3: TO-PRO-3 nuclear staining. Scale bars: 50μm.
(TIF)

S6 Fig TUNEL assay and P-Thr of discs with simultaneous *Sod1*:*Cat* and *rpr* activation (*sal*^*E/Pv*^*>rpr*, *Sod1*:*Cat;* low oxidative stress). Purple area shows where transgenes were activated.
(TIF)

S7 Fig Supporting information for Fig 5. (A) P-JNK staining in wild type, *sal*^*E/Pv*^*>rpr*, and two examples of *sal*^*E/Pv*^*>rpr ci>Ask1*^*RNAi*^. Thermal LUT was used to detect pixel differences. TP-3: TO-PRO-3 nuclear staining. Purple indicates the dead zone. Colorized red indicates the *ci>Ask1*^*RNAi*^ zone. (B) Quantification of the P-JNK in raw images. A and P indicate the approximate areas measured for pixel intensity in n = 15 for each genotype. (C-E) Live imaging of a *puc-Gal4*, *UAS-GFP* used as a reporter of the JNK pathway. Discs were cut and cultured for 6 hours. White lines indicate the wound site. White arrows point to the endogenous *puc* expression zone at the tip of the notum. (C) Wild type. (D) *Ask1*^*MB06487*^ heterozygous. The nuclear marker NucRed Live647 was added in the culture medium. Scale bar: 50μm. (E) Quantification of *puc-Gal4*, *UAS-GFP* reporter at the wound site (n = 13 discs each genotype). Error bars indicate standard deviation. **P<0.01, ***P<0.001.
(TIF)

S1 Appendix Computational verification that human Ser83 domain is missing on *Drosophila melanogaster* Ask1 gene loci. This file contains the plain text genewise alignment of the human M3K5_HUMAN protein sequence against the genomic segment of 5kbp upstream/downstream that is centered on the annotated fly Ask1 gene transcription start site. No alignment was reported for the first 126 residues of the human protein where the Ser83 RxRxx[ST] domain has been described.
(PDF)

S2 Appendix Complete alignment of the selected 50 MAP3K5/Ask1 protein orthologs that illustrates high conservation across the sequence. Many positions are preserved from sponges to humans, including serine/threonine residues, whilst other show a dual pattern of conservation with specific substitutions between chordate and invertebrate species. From the whole set of 338 sequences on 164 species that define the EggNOG (http://eggnogdb.embl.de) homologs cluster KOG4279, comprising human MAP3K5 and its counterpart in fly Ask1-PC, we filtered out 49 proteins that were confidently annotated as MAP3K5 orthologs on different taxa plus fly Ask1-PB isoform downloaded from FlyBase, which are listed here (names shown in parentheses are those used on the alignment sequence labels and refer to UniProt identifiers when possible): *Homo sapiens* ENSP00000351908 (M3K5_HUMAN), *Pan troglodytes* ENSPTRP00000043317 (K7CFQ9_PANTR), *Gorilla gorilla* ENSGGOP00000022651 (G3S3K1_GORGO), *Macaca mulatta* ENSMMUP00000021508 (F7G2W1_MACMU), Pongo abelii ENSPPYP00000019077 (H2PKF0_PONAB), *Otolemur garnettii* ENSOGAP00000000498 (H0WGN9_OTOGA), *Callithrix jacchus* ENSCJAP00000015659 (F7GZH9_CALJA), *Rattus norvegicus* ENSRNOP00000051338 (D3ZW27_RAT), *Mus musculus* ENSMUSP00000093485 (M3K5_MOUSE), *Cavia porcellus* ENSCPOP00000003829 (H0V2Q4_CAVPO), *Bos taurus* ENSBTAP00000011949 (F1MXH6_BOVIN), *Pteropus vampyrus* ENSPVAP00000009127 (ens9127_PTEVA), *Mustela putorius furo* ENSMPUP00000014726 (G9K9I6_MUSPF), *Loxodonta africana* ENSLAFP00000000494 (G3UDL3_LOXAF), *Echinops telfairi* ENSETEP00000012543 (ens12543_ECHTE), *Canis lupus familiaris* ENSCAFP00000000362 (F1PA02_CANFA), *Ailuropoda melanoleuca* ENSAMEP00000010579 (D2H0V2_AILME), *Tursiops truncatus* ENSTTRP00000002388 (ens2388_TURTR), *Dasypus novemcinctus* ENSDNOP00000014966 (ens14966_DASNO), *Sarcophilus harrisii* ENSSHAP00000014955 (G3WHQ0_SARHA), *Gallus gallus* ENSGALP00000022482 (F1NYS9_CHICK), *Meleagris gallopavo* ENSMGAP00000013731 (G1NK21_MELGA), *Taeniopygia guttata* ENSTGUP00000011202 (H0ZKT1_TAEGU), *Pelodiscus sinensis* ENSPSIP00000018784 (K7GES3_PELSI), *Xenopus tropicalis* ENSXETP00000005271 (F7A415_XENT), *Gasterosteus aculeatus* ENSGACP00000009628 (G3NWA9_GASAC), *Oreochromis niloticus* ENSONIP00000007016 (I3JDS6_ORENI), *Danio rerio* ENSDARP00000046640 (E7F2L3_DANRE), *Takifugu rubripes* ENSTRUP00000002505 (H2RQT9_TAKRU), *Tetraodon nigroviridis* ENSTNIP00000022322 (Q4RFA2_TETNG), *Branchiostoma floridae* JGI219597 (C3XTE7_BRAFL), *Drosophila melanogaster* FBpp0089256 (Ask1-PB_DROME) and FBpp0289484 (Ask1-PC_DROME), *D*. *ananassae* FBpp0120816 (B3LXS9_DROAN), *Culex quinquefasciatus* CPIJ008487-PA (B0WN02_CULQU), *Anopheles gambiae* AGAP000747-PB (QEA6_ANOGA), *Aedes aegypti* AAEL008311-PA (Q16Z55_AEDAE), *Bombyx mori* BGIBMGA010545-TA (E9JEG8_BOMMO), *Danaus plexippus* EHJ64716 (G6DP69_DANPL), *Apis mellifera* GB14238-PA (H9KAX6_APIME), *Nasonia vitripennis* NV13729-PA (K7IXA9_NASVI), *Acyrthosiphon pisum* ACYPI009039-PA (J9K801_ACYPI), *Tribolium castaneum* TC000430-PA (TCOGS2_TRICA), *Daphnia pulex* DappuP328832 (P32883_DAPPU), *Caenorhabditis elegans* F59A6.1 (NSY-1_CAEEL), *C*. *japonica* CJA14063 (K7I1N3_CAEJA), *C*. *briggsae* CBG02299 (A8WUN1_CAEBR), Strongylocentrotus purpuratus SPU_010872tr (H3I9Y9_STRPU), *Amphimedon queenslandica* PAC_15725247 (15725247_AMPQE), *Trichoplax adhaerens* TriadP10277 (B3RYN2_TRIAD).
(PDF)

S3 Appendix Data for the graphics of Fig 4. (DOCX)

## References

[1] Hariharan IK, Serras F. Imaginal disc regeneration takes flight. Curr Opin Cell Biol. 2017;48 10.1016/j.ceb.2017.03.005 28376317 PMC5591769

[2] Sen CK, Roy S. Redox signals in wound healing. Biochim Biophys Acta. 2008;1780: 1348–61. 10.1016/j.bbagen.2008.01.006 18249195 PMC2574682

[3] Veal EA, Day AM, Morgan BA. Hydrogen peroxide sensing and signaling. Mol Cell. 2007;26: 1–14. 10.1016/j.molcel.2007.03.016 17434122

[4] Serras F. The benefits of oxidative stress for tissue repair and regeneration. Fly (Austin). 2016;10 10.1080/19336934.2016.1188232 27172271 PMC4970533

[5] Santabárbara-Ruiz P, López-Santillán M, Martínez-Rodríguez I, Binagui-Casas A, Pérez L, Milán M, et al ROS-Induced JNK and p38 Signaling Is Required for Unpaired Cytokine Activation during Drosophila Regeneration. PLoS Genet. 2015;11: e1005595 10.1371/journal.pgen.1005595 26496642 PMC4619769

[6] Fogarty CE, Diwanji N, Lindblad JL, Tare M, Amcheslavsky A, Makhijani K, et al Extracellular Reactive Oxygen Species Drive Apoptosis-Induced Proliferation via Drosophila Macrophages. Curr Biol. 2016; 10.1016/j.cub.2015.12.064 26898463 PMC4765900

[7] Khan SJ, Abidi SNF, Skinner A, Tian Y, Smith-Bolton RK. The Drosophila Duox maturation factor is a key component of a positive feedback loop that sustains regeneration signaling. PLoS Genet. 2017;13 10.1371/journal.pgen.1006937 28753614 PMC5550008

[8] Finkel T. Signal transduction by reactive oxygen species. J Cell Biol. 2011;194: 7–15. 10.1083/jcb.201102095 21746850 PMC3135394

[9] Lee N, Maurange C, Ringrose L, Paro R. Suppression of Polycomb group proteins by JNK signalling induces transdetermination in Drosophila imaginal discs. Nature. 2005;438: 234–237. nature04120 [pii] 10.1038/nature04120 16281037

[10] Herrera SC, Martin R, Morata G. Tissue homeostasis in the wing disc of Drosophila melanogaster: immediate response to massive damage during development. PLoS Genet. 2013;9: e1003446 10.1371/journal.pgen.1003446 23633961 PMC3636033

[11] Smith-Bolton RK, Worley MI, Kanda H, Hariharan IK. Regenerative growth in Drosophila imaginal discs is regulated by Wingless and Myc. Dev Cell. 2009;16: 797–809. 10.1016/j.devcel.2009.04.015 19531351 PMC2705171

[12] Ryoo HD, Gorenc T, Steller H. Apoptotic cells can induce compensatory cell proliferation through the JNK and the Wingless signaling pathways. Dev Cell. 2004;7: 491–501. S1534580704003247 [pii] 10.1016/j.devcel.2004.08.019 15469838

[13] Bosch M, Serras F, Martin-Blanco E, Baguna J, Martín-Blanco E, Baguñà J. JNK signaling pathway required for wound healing in regenerating Drosophila wing imaginal discs. Dev Biol. 2005;280: 73–86. S0012-1606(05)00003-5 [pii] 10.1016/j.ydbio.2005.01.002 15766749

[14] Mattila J, Omelyanchuk L, Kyttala S, Turunen H, Nokkala S. Role of Jun N-terminal Kinase (JNK) signaling in the wound healing and regeneration of a Drosophila melanogaster wing imaginal disc. Int J Dev Biol. 2005;49: 391–399. 052006jm [pii] 10.1387/ijdb.052006jm 15968584

[15] Pastor-Pareja JC, Wu M, Xu T. An innate immune response of blood cells to tumors and tissue damage in Drosophila. Dis Model Mech. 2008;1: 144–154. 10.1242/dmm.000950 19048077 PMC2562178

[16] Fan Y, Wang S, Hernandez J, Yenigun VB, Hertlein G, Fogarty CE, et al Genetic models of apoptosis-induced proliferation decipher activation of JNK and identify a requirement of EGFR signaling for tissue regenerative responses in Drosophila. PLoS Genet. Public Library of Science; 2014;10: e1004131 10.1371/journal.pgen.1004131 24497843 PMC3907308

[17] Galko MJ, Krasnow MA. Cellular and genetic analysis of wound healing in Drosophila larvae. PLoS Biol. 2004;2: E239 10.1371/journal.pbio.0020239 15269788 PMC479041

[18] Harris RE, Setiawan L, Saul J, Hariharan IK. Localized epigenetic silencing of a damage-activated WNT enhancer limits regeneration in mature Drosophila imaginal discs. Elife. 2016;5 10.7554/eLife.11588 26840050 PMC4786413

[19] Repiso A, Bergantiños C, Corominas M, Serras F. Tissue repair and regeneration in Drosophila imaginal discs. Dev Growth Differ. 2011;53 10.1111/j.1440-169X.2010.01247.x 21338344

[20] Repiso A, Bergantiños C, Serras F. Cell fate respecification and cell division orientation drive intercalary regeneration in Drosophila wing discs. Dev. 2013;140 10.1242/dev.095760 23903186

[21] Seisenbacher G, Hafen E, Stocker H. MK2-dependent p38b signalling protects Drosophila hindgut enterocytes against JNK-induced apoptosis under chronic stress. PLoS Genet. 2011;7: e1002168 10.1371/journal.pgen.1002168 21829386 PMC3150449

[22] Chakrabarti S, Poidevin M, Lemaitre B. The Drosophila MAPK p38c regulates oxidative stress and lipid homeostasis in the intestine. PLoS Genet. Public Library of Science; 2014;10: e1004659 10.1371/journal.pgen.1004659 25254641 PMC4177744

[23] Craig CR, Fink JL, Yagi Y, Ip YT, Cagan RL. A Drosophila p38 orthologue is required for environmental stress responses. EMBO Rep. 2004;5: 1058–63. 10.1038/sj.embor.7400282 15514678 PMC1299177

[24] Vrailas-Mortimer A, del Rivero T, Mukherjee S, Nag S, Gaitanidis A, Kadas D, et al A Muscle-Specific p38 MAPK/Mef2/MnSOD Pathway Regulates Stress, Motor Function, and Life Span in Drosophila. Dev Cell. 2011;21: 783–795. 10.1016/j.devcel.2011.09.002 22014527 PMC3199449

[25] Worley MI, Alexander LA, Hariharan IK. CtBP impedes JNK- and Upd/STAT-driven cell fate misspecifications in regenerating Drosophila imaginal discs. Elife. 2018;7 10.7554/eLife.30391 29372681 PMC5823544

[26] Hunter M V., Willoughby PM, Bruce AEE, Fernandez-Gonzalez R. Oxidative Stress Orchestrates Cell Polarity to Promote Embryonic Wound Healing. Dev Cell. 2018;47: 377–387.e4. 10.1016/j.devcel.2018.10.013 30399336

[27] Brock AR, Seto M, Smith-Bolton RK. Cap-n-Collar Promotes Tissue Regeneration by Regulating ROS and JNK Signaling in the Drosophila melanogaster Wing Imaginal Disc. Genetics. 2017;206: 1505–1520. 10.1534/genetics.116.196832 28512185 PMC5500147

[28] Takatsu Y, Nakamura M, Stapleton M, Danos MC, Matsumoto K, O’Connor MB, et al TAK1 participates in c-Jun N-terminal kinase signaling during Drosophila development. Mol Cell Biol. 2000;20: 3015–26. 10757786 10.1128/mcb.20.9.3015-3026.2000 PMC85571

[29] Adachi-Yamada T, Fujimura-Kamada K, Nishida Y, Matsumoto K. Distortion of proximodistal information causes JNK-dependent apoptosis in Drosophila wing. Nature. Division of Biological Science, Graduate School of Science, Nagoya University, Japan Science and Technology Corporation. adachi@bio.nagoya-u.ac.jp; 1999;400: 166–169. 10.1038/22112 10408443

[30] Glise B, Bourbon H, Noselli S. hemipterous encodes a novel Drosophila MAP kinase kinase, required for epithelial cell sheet movement. Cell. 1995;83: 451–461. 0092-8674(95)90123-X [pii] 8521475 10.1016/0092-8674(95)90123-x

[31] Shlevkov E, Morata G. A dp53/JNK-dependant feedback amplification loop is essential for the apoptotic response to stress in Drosophila. Cell Death Differ. 2012;19: 451–60. 10.1038/cdd.2011.113 21886179 PMC3278728

[32] Takeda K, Noguchi T, Naguro I, Ichijo H. Apoptosis Signal-Regulating Kinase 1 in Stress and Immune Response. Annu Rev Pharmacol Toxicol. 2008;48: 199–225. 10.1146/annurev.pharmtox.48.113006.094606 17883330

[33] Ichijo H, Nishida E, Irie K, ten Dijke P, Saitoh M, Moriguchi T, et al Induction of apoptosis by ASK1, a mammalian MAPKKK that activates SAPK/JNK and p38 signaling pathways. Science. 1997;275: 90–4. 8974401 10.1126/science.275.5296.90

[34] Wang XS, Diener K, Jannuzzi D, Trollinger D, Tan TH, Lichenstein H, et al Molecular cloning and characterization of a novel protein kinase with a catalytic domain homologous to mitogen-activated protein kinase kinase kinase. J Biol Chem. 1996;271: 31607–11. 8940179 10.1074/jbc.271.49.31607

[35] Saitoh M, Nishitoh H, Fujii M, Takeda K, Tobiume K, Sawada Y, et al Mammalian thioredoxin is a direct inhibitor of apoptosis signal-regulating kinase (ASK) 1. EMBO J. 1998;17: 2596–2606. 10.1093/emboj/17.9.2596 9564042 PMC1170601

[36] Liu H, Nishitoh H, Ichijo H, Kyriakis JM. Activation of apoptosis signal-regulating kinase 1 (ASK1) by tumor necrosis factor receptor-associated factor 2 requires prior dissociation of the ASK1 inhibitor thioredoxin. Mol Cell Biol. 2000;20: 2198–208. 10688666 10.1128/mcb.20.6.2198-2208.2000 PMC110836

[37] Sakauchi C, Wakatsuki H, Ichijo H, Hattori K. Pleiotropic properties of ASK1. Biochim Biophys Acta—Gen Subj. 2017;1861: 3030–3038. 10.1016/j.bbagen.2016.09.028 27693599

[38] Kim AH, Khursigara G, Sun X, Franke TF, Chao M V. Akt Phosphorylates and Negatively Regulates Apoptosis Signal-Regulating Kinase 1. Mol Cell Biol. 2001;21: 893–901. 10.1128/MCB.21.3.893-901.2001 11154276 PMC86680

[39] Hietakangas V, Cohen SM. Regulation of Tissue Growth through Nutrient Sensing. 2009; 10.1146/annurev-genet-102108-134815 19694515

[40] Hariharan IK, Bilder D. Regulation of imaginal disc growth by tumor-suppressor genes in Drosophila. Annu Rev Genet. 2006;40: 335–61. 10.1146/annurev.genet.39.073003.100738 16872256

[41] Song MS, Salmena L, Pandolfi PP. The functions and regulation of the PTEN tumour suppressor. Nat Rev Mol Cell Biol. Nature Publishing Group; 2012;13: 283–296. 10.1038/nrm3330 22473468

[42] Böhni R, Riesgo-Escovar J, Oldham S, Brogiolo W, Stocker H, Andruss BF, et al Autonomous control of cell and organ size by CHICO, a Drosophila homolog of vertebrate IRS1-4. Cell. 1999;97: 865–75. 10399915 10.1016/s0092-8674(00)80799-0

[43] Gao X, Neufeld TP, Pan D. Drosophila PTEN Regulates Cell Growth and Proliferation through PI3K-Dependent and -Independent Pathways. Dev Biol. 2000;221: 404–418. 10.1006/dbio.2000.9680 10790335

[44] Huang H, Potter CJ, Tao W, Li DM, Brogiolo W, Hafen E, et al PTEN affects cell size, cell proliferation and apoptosis during Drosophila eye development. Development. 1999;126: 5365–72. 10556061 10.1242/dev.126.23.5365

[45] Goberdhan DC, Paricio N, Goodman EC, Mlodzik M, Wilson C. Drosophila tumor suppressor PTEN controls cell size and number by antagonizing the Chico/PI3-kinase signaling pathway. Genes Dev. 1999;13: 3244–58. 10617573 10.1101/gad.13.24.3244 PMC317204

[46] Bergantiños C, Corominas M, Serras F. Cell death-induced regeneration in wing imaginal discs requires JNK signalling. Development. 2010;137: 1169–1179. 137/7/1169 [pii] 10.1242/dev.045559 20215351

[47] Yagi R, Mayer F, Basler K. Refined LexA transactivators and their use in combination with the Drosophila Gal4 system. Proc Natl Acad Sci U S A. Institute of Molecular Life Sciences, University of Zurich, CH-8057 Zurich, Switzerland.; 2010;107: 16166–16171. 1005957107 [pii] 10.1073/pnas.1005957107 20805468 PMC2941298

[48] Kuranaga E, Kanuka H, Igaki T, Sawamoto K, Ichijo H, Okano H, et al Reaper-mediated inhibition of DIAP1-induced DTRAF1 degradation results in activation of JNK in Drosophila. Nat Cell Biol. 2002;4: 705–710. 10.1038/ncb842 12198495

[49] Cho Y-C, Park JE, Park BC, Kim J-H, Jeong DG, Park SG, et al Cell cycle-dependent Cdc25C phosphatase determines cell survival by regulating apoptosis signal-regulating kinase 1. Cell Death Differ. Nature Publishing Group; 2015;22: 1605–1617. 10.1038/cdd.2015.2 25633196 PMC4563786

[50] Stocker H, Andjelkovic M, Oldham S, Laffargue M, Wymann MP, Hemmings BA, et al Living with lethal PIP3 levels: viability of flies lacking PTEN restored by a PH domain mutation in Akt/PKB. Science. 2002;295: 2088–91. 10.1126/science.1068094 11872800

[51] Staveley BE, Ruel L, Jin J, Stambolic V, Mastronardi FG, Heitzler P, et al Genetic analysis of protein kinase B (AKT) in Drosophila. Curr Biol. 1998;8: 599–602. 9601646 10.1016/s0960-9822(98)70231-3

[52] Nishitoh H, Matsuzawa A, Tobiume K, Saegusa K, Takeda K, Inoue K, et al ASK1 is essential for endoplasmic reticulum stress-induced neuronal cell death triggered by expanded polyglutamine repeats. Genes Dev. Cold Spring Harbor Laboratory Press; 2002;16: 1345–55. 10.1101/gad.992302 12050113 PMC186318

[53] Zhuang Z-H, Zhou Y, Yu M-C, Silverman N, Ge B-X. Regulation of Drosophila p38 activation by specific MAP2 kinase and MAP3 kinase in response to different stimuli. Cell Signal. 2006;18: 441–448. 10.1016/j.cellsig.2005.05.013 16014325

[54] Uhlirova M, Bohmann D. JNK- and Fos-regulated Mmp1 expression cooperates with Ras to induce invasive tumors in Drosophila. EMBO J. Department of Biomedical Genetics, University of Rochester Medical Center, Rochester, NY 14642, USA.; 2006;25: 5294–5304. 7601401 [pii] 10.1038/sj.emboj.7601401 17082773 PMC1636619

[55] Haynie J, Bryant PJ. The effects of X-rays on the proliferation dynamics of cells in the imaginal wing disc of Drosophila melanogaster. Roux’s Arch Dev Biol. 1977;183: 85–100. 10.1007/BF00848779 28304897

[56] Bergmann A, Steller H. Apoptosis, stem cells, and tissue regeneration. Sci Signal. 2010;3: re8 10.1126/scisignal.3145re8 20978240 PMC2991142

[57] Ferreira F, Luxardi G, Reid B, Zhao M. Early bioelectric activities mediate redox-modulated regeneration. Development. 2016;143: 4582–4594. 10.1242/dev.142034 27827821 PMC5201032

[58] Zhang Q, Wang Y, Man L, Zhu Z, Bai X, Wei S, et al Reactive oxygen species generated from skeletal muscles are required for gecko tail regeneration. Sci Rep. Nature Publishing Group; 2016;6: 20752 10.1038/srep20752 26853930 PMC4745102

[59] Pirotte N, Stevens A-S, Fraguas S, Plusquin M, Van Roten A, Van Belleghem F, et al Reactive Oxygen Species in Planarian Regeneration: An Upstream Necessity for Correct Patterning and Brain Formation. Oxid Med Cell Longev. Hindawi Publishing Corporation; 2015;2015: 392476 10.1155/2015/392476 26180588 PMC4477255

[60] Wenger Y, Buzgariu W, Reiter S, Galliot B. Injury-induced immune responses in Hydra. Semin Immunol. 2014;26: 277–294. 10.1016/j.smim.2014.06.004 25086685

[61] Moreira S, Stramer B, Evans I, Wood W, Martin P. Prioritization of competing damage and developmental signals by migrating macrophages in the Drosophila embryo. Curr Biol. 2010;20: 464–70. 10.1016/j.cub.2010.01.047 20188558

[62] Yoo SK, Starnes TW, Deng Q, Huttenlocher A. Lyn is a redox sensor that mediates leukocyte wound attraction in vivo. Nature. NIH Public Access; 2011;480: 109–12. 10.1038/nature10632 22101434 PMC3228893

[63] Yoo SK, Freisinger CM, LeBert DC, Huttenlocher A. Early redox, Src family kinase, and calcium signaling integrate wound responses and tissue regeneration in zebrafish. J Cell Biol. The Rockefeller University Press; 2012;199: 225–34. 10.1083/jcb.201203154 23045550 PMC3471241

[64] Gauron C, Rampon C, Bouzaffour M, Ipendey E, Teillon J, Volovitch M, et al Sustained production of ROS triggers compensatory proliferation and is required for regeneration to proceed. Sci Rep. 2013;3: 2084 10.1038/srep02084 23803955 PMC3694286

[65] Han P, Zhou X-H, Chang N, Xiao C-L, Yan S, Ren H, et al Hydrogen peroxide primes heart regeneration with a derepression mechanism. Cell Res. Nature Publishing Group; 2014;24: 1091–107. 10.1038/cr.2014.108 25124925 PMC4152734

[66] Niethammer P, Grabher C, Look AT, Mitchison TJ. A tissue-scale gradient of hydrogen peroxide mediates rapid wound detection in zebrafish. Nature. 2009;459: 996–9. 10.1038/nature08119 19494811 PMC2803098

[67] Love NR, Chen Y, Ishibashi S, Kritsiligkou P, Lea R, Koh Y, et al Amputation-induced reactive oxygen species are required for successful Xenopus tadpole tail regeneration. Nat Cell Biol. 2013;15: 222–8. 10.1038/ncb2659 23314862 PMC3728553

[68] Wells BS, Yoshida E, Johnston LA. Compensatory proliferation in Drosophila imaginal discs requires Dronc-dependent p53 activity. Curr Biol. 2006;16: 1606–1615. S0960-9822(06)01913-0 [pii] 10.1016/j.cub2006.07.046 16920621 PMC1764442

[69] Katsuyama T, Comoglio F, Seimiya M, Cabuy E, Paro R. During Drosophila disc regeneration, JAK/STAT coordinates cell proliferation with Dilp8-mediated developmental delay. Proc Natl Acad Sci U S A. 2015;112: E2327–36. 10.1073/pnas.1423074112 25902518 PMC4426433

[70] Verghese S, Su TT. Drosophila Wnt and STAT Define Apoptosis-Resistant Epithelial Cells for Tissue Regeneration after Irradiation. Edgar BA, editor. PLoS Biol. 2016;14: e1002536 10.1371/journal.pbio.1002536 27584613 PMC5008734

[71] La Fortezza M, Schenk M, Cosolo A, Kolybaba A, Grass I, Classen A-K. JAK/STAT signalling mediates cell survival in response to tissue stress. Development. 2016;143: 2907–2919. 10.1242/dev.132340 27385008

[72] Ahmed-de-Prado S, Diaz-Garcia S, Baonza A. JNK and JAK/STAT signalling are required for inducing loss of cell fate specification during imaginal wing discs regeneration in Drosophila melanogaster. Dev Biol. 2018;441: 31–41. 10.1016/j.ydbio.2018.05.021 29870691

[73] McClure KD, Sustar A, Schubiger G. Three genes control the timing, the site and the size of blastema formation in Drosophila. Dev Biol. University of California, San Francisco, Department of Anatomy, 1550 4th Street, Rock Hall, Mail Code 2822, San Francisco, CA 94158, USA. kimberly.mcclure@ucsf.edu; 2008;319: 68–77. S0012-1606(08)00267-4 [pii] 10.1016/j.ydbio.2008.04.004 18485344 PMC2483308

[74] Sun G, Irvine KD. Regulation of Hippo signaling by Jun kinase signaling during compensatory cell proliferation and regeneration, and in neoplastic tumors. Dev Biol. Howard Hughes Medical Institute, Waksman Institute and Department of Molecular Biology and Biochemistry, Rutgers, The State University of New Jersey, Piscataway, NJ 08854, USA.: Elsevier Inc; 2011;350: 139–151. 10.1016/j.ydbio.2010.11.036 21145886 PMC3038240

[75] Meserve JH, Duronio RJ. Scalloped and Yorkie are required for cell cycle re-entry of quiescent cells after tissue damage. Development. 2015;142: 2740–51. 10.1242/dev.119339 26160905 PMC4550963

[76] Repiso A, Bergantinos C, Serras F, Bergantiños C, Serras F. Cell fate respecification and cell division orientation drive intercalary regeneration in Drosophila wing discs. Development. 2013;140: 3541–3551. 10.1242/dev.095760 23903186

[77] Kamata H, Honda S-I, Maeda S, Chang L, Hirata H, Karin M. Reactive oxygen species promote TNFalpha-induced death and sustained JNK activation by inhibiting MAP kinase phosphatases. Cell. 2005;120: 649–61. 10.1016/j.cell.2004.12.041 15766528

[78] Leslie NR, Bennett D, Lindsay YE, Stewart H, Gray A, Downes CP. Redox regulation of PI 3-kinase signalling via inactivation of PTEN. EMBO J. European Molecular Biology Organization; 2003;22: 5501–10. 10.1093/emboj/cdg513 14532122 PMC213768

[79] Agrawal N, Delanoue R, Mauri A, Basco D, Pasco M, Thorens B, et al The Drosophila TNF Eiger Is an Adipokine that Acts on Insulin-Producing Cells to Mediate Nutrient Response. Cell Metab. 2016;23: 675–684. 10.1016/j.cmet.2016.03.003 27076079

[80] Birney E, Clamp M, Durbin R. GeneWise and Genomewise. Genome Res. 2004;14: 988–995. 10.1101/gr.1865504 15123596 PMC479130

[81] The UniProt Consortium. UniProt: the universal protein knowledgebase. Nucleic Acids Res. 2017;45: D158–D169. 10.1093/nar/gkw1099 27899622 PMC5210571

[82] Gramates LS, Marygold SJ, Santos G Dos, Urbano J-M, Antonazzo G, Matthews BB, et al FlyBase at 25: looking to the future. Nucleic Acids Res. 2017;45: D663–D671. 10.1093/nar/gkw1016 27799470 PMC5210523

[83] Finn RD, Coggill P, Eberhardt RY, Eddy SR, Mistry J, Mitchell AL, et al The Pfam protein families database: towards a more sustainable future. Nucleic Acids Res. 2016;44: D279–85. 10.1093/nar/gkv1344 26673716 PMC4702930

[84] Huerta-Cepas J, Szklarczyk D, Forslund K, Cook H, Heller D, Walter MC, et al eggNOG 4.5: a hierarchical orthology framework with improved functional annotations for eukaryotic, prokaryotic and viral sequences. Nucleic Acids Res. 2016;44: D286–93. 10.1093/nar/gkv1248 26582926 PMC4702882

[85] Katoh K, Standley DM. MAFFT multiple sequence alignment software version 7: improvements in performance and usability. Mol Biol Evol. 2013;30: 772–80. 10.1093/molbev/mst010 23329690 PMC3603318

[86] Beitz E. TEXshade: shading and labeling of multiple sequence alignments using LATEX2 epsilon. Bioinformatics. 2000;16: 135–9. 10842735 10.1093/bioinformatics/16.2.135

