# Supplementary figures and images for: Ask1 and Akt act synergistically to promote ROS-dependent regeneration in Drosophila
Source: PLoS Genet. 2019 Jan 24;15(1):e1007926. doi: 10.1371/journal.pgen.1007926 (PMC6363233; doi:10.1371/journal.pgen.1007926)

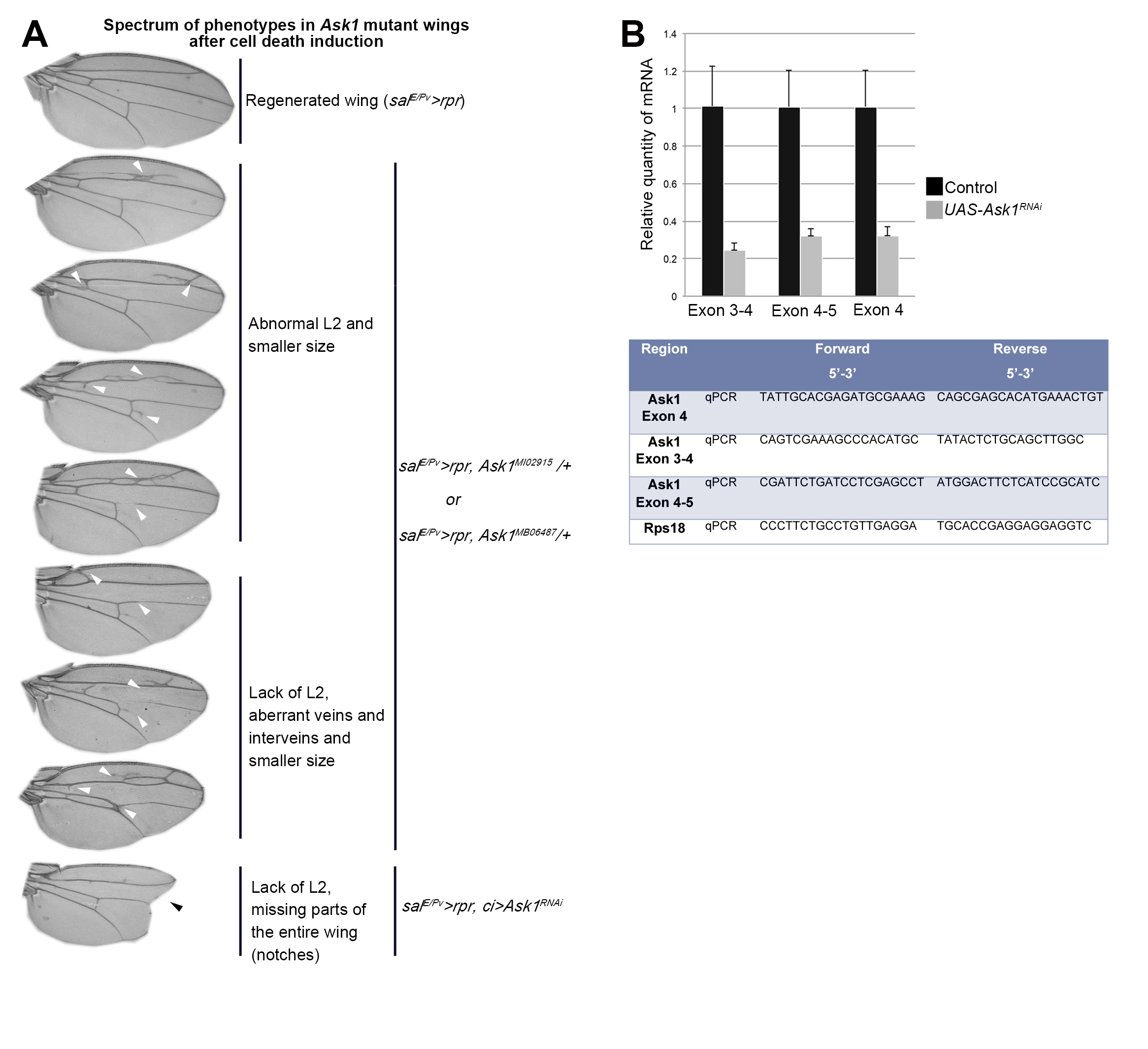

Supplement: S1 Fig — (A) Spectrum of wings with anomalous regeneration, ranging from complete regeneration (salE/Pv>rpr in wild type, top) to a severe effect found after Ask1RNAi (salE/Pv>rpr in mutant conditions, bottom). It includes a range of defects found after inducing cell death in Ask1 heterozygous background and after UAS-Ask1RNAi. Arrowheads indicate some defects. (B) Real time quantitative PCR of the UAS-Ask1RNAi. The table indicates the primers used. The RT-qPCR analysis is shown for Ask1 expression in wing discs of controls (hh>RFP) and Ask1RNAi (hh>UAS-Ask1RNAi). qPCR results are presented as relative quantity of mRNA between control and Ask1RNAi. Error bars represent the standard error of the mean from three biological replicates (n = 40 discs, each replicate). (TIF) [file pgen.1007926.s001.tif]

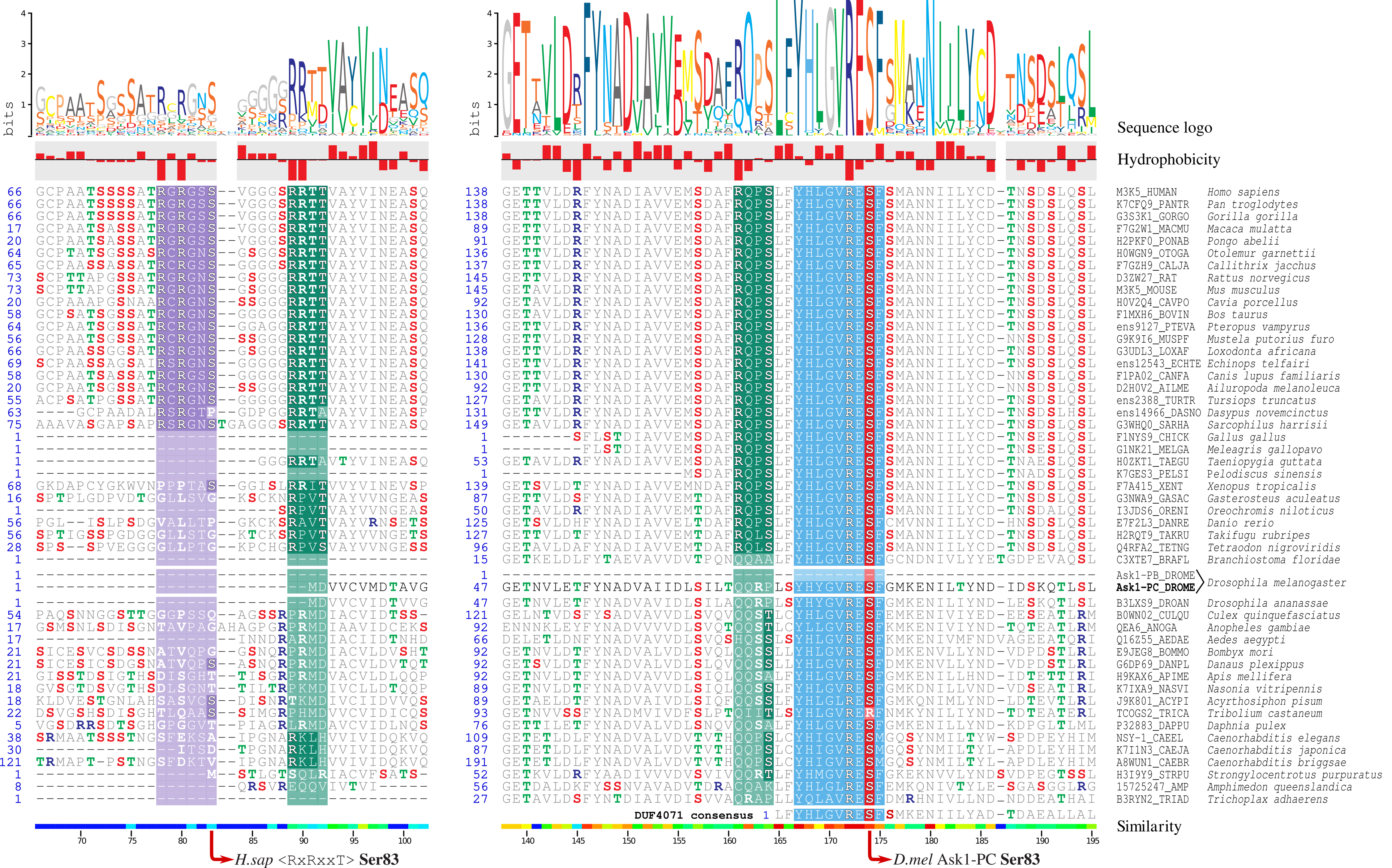

Supplement: S2 Fig — Two blocks of conserved columns pinpointing the aforementioned residues were selected from the whole-length alignment of 50 MAP3K5/Ask1 protein orthologs provided on the S2 Appendix. From top to bottom: sequence logos displaying information content based on amino acid frequencies; an estimate of hydrophobicity of the amino acid residues; the aligned sequences split into chordate and invertebrate sequences, above or below the two Drosophila melanogaster Ask1 isoforms respectively; finally, the similarity is depicted as a color gradient ranging from low (blue) to high (red). Bottom reference coordinates for the aligned residues are based on human reference sequence, which is listed on the first row. Serines, threonines, and arginines were labeled in red, green and blue when appearing outside the domain blocks, but shown in white and boldface within those blocks. Numbers on the left side of the alignment blocks correspond to the relative position of the first amino acid within those blocks for each species sequences. Left alignment block contains a domain in purple that matches the consensus RxRxx[ST] pattern, although conservation is limited to birds and mammals (dark versus light shades of violet). On the right, the domain highlighted in cyan is conserved all across the diverse taxa, from human (top) to sponges (bottom), and it contains a serine (red background) that corresponds to the fly Ser83. On both panels, another domain matching a degenerated xxRxx[ST] pattern that is only conserved within vertebrate sequences is shown as a green block. Right alignment also includes at the bottom the consensus sequence of a PFAM domain of unknown function (DUF4071) that starts two residues before the Ser83 conserved block; this block within the domain shows high conservation across the different protein families considered when building the corresponding model. (TIF) [file pgen.1007926.s002.tif]

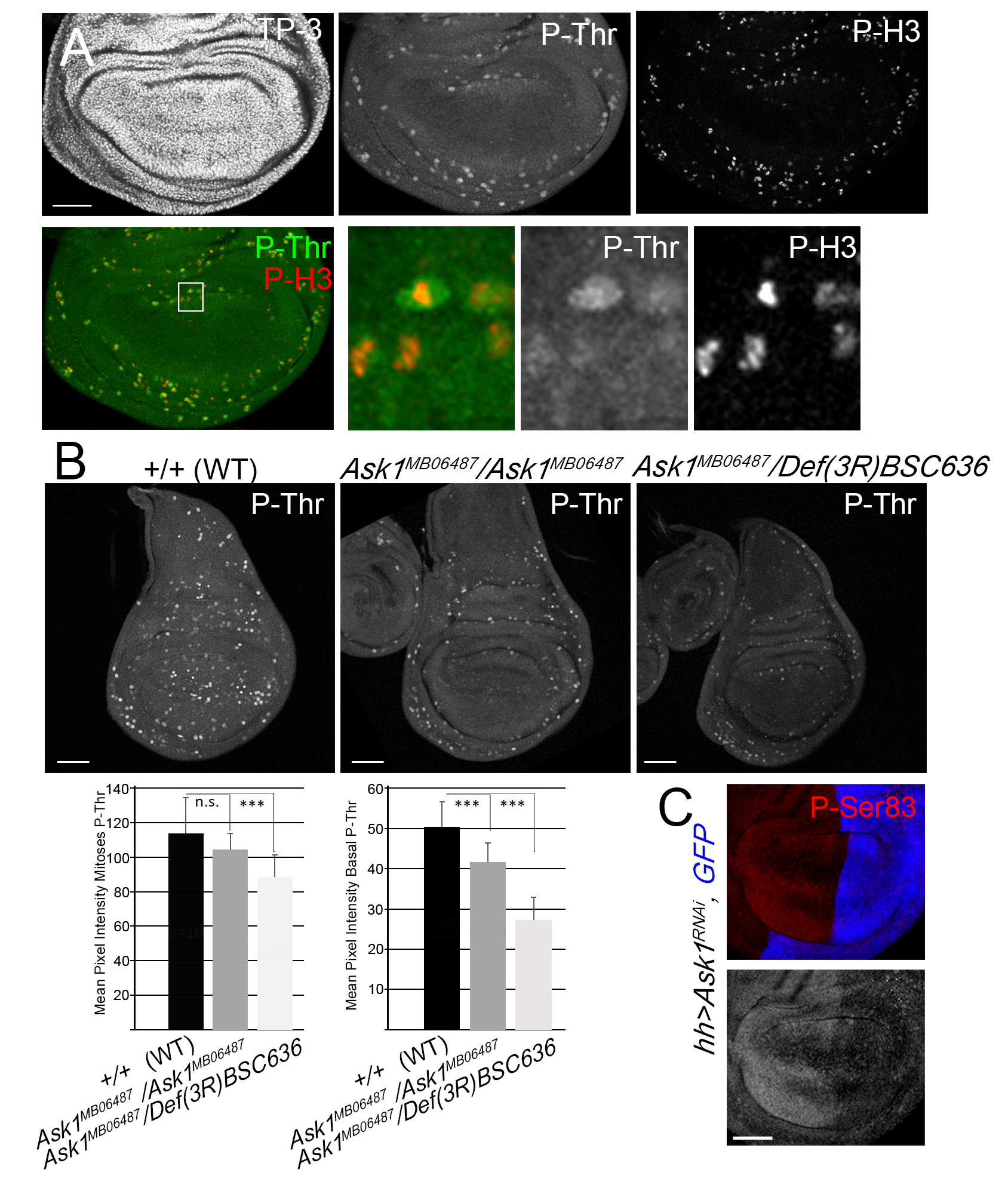

Supplement: S3 Fig — (A) Mitotic (P-H3 positive) and Ask1 P-Thr. Lower right images: Zoom of mitotic cells from the white square. TP-3: TO-PRO-3 nuclear staining. (B) The basal levels of P-Thr in wild-type (WT) discs (mitotic and interphase cells) decrease in the Ask1MB06487 homozygous mutant background and are strongly reduced in the Ask1MB06487 mutant background over the deficiency Def(3R)50636. Quantification of mean pixel intensity for P-Thr in mitotic and for P-Thr in non-mitotic (basal levels) cells are shown in separate graphics. P-Thr mean Pixel intensity in mitotic cells of the wild-type discs (+/+), 113.92±20.60, S.D. (n = 10); of the Ask1MB06487/Ask1MB06487 discs, 104.43±9.54, S.D. (n = 10); and of the Ask1MB06487/Def(3R)BSC636 88.37±13.03, S.D. (n = 10). Mean pixel intensity of the basal levels in interphase cells of the wild-type, 50.44±6.27, S.D. (n = 20); of the Ask1MB06487/Ask1MB06487, 41.65±4.80, S.D. (n = 10); and of the Ask1MB06487/Def(3R)BSC636, 27.22±5.73, S.D. (n = 18). (C) Inhibition of the endogenous Ask1 P-Ser83 levels after expressing the UAS-Ask1RNAi in the posterior compartment, using the hh-Gal4 driver. Scale bars: 5μm. (TIF) [file pgen.1007926.s003.tif]

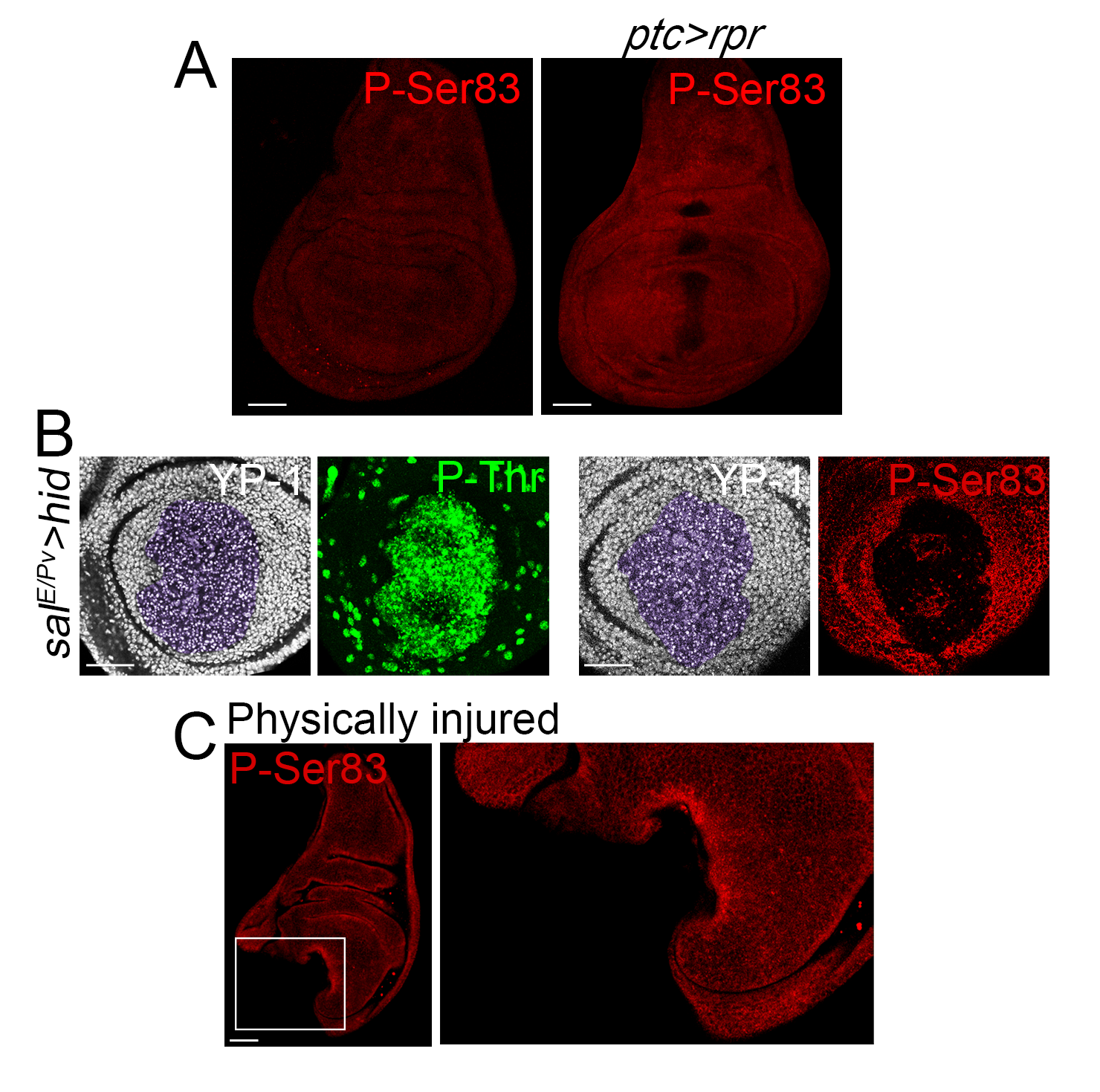

Supplement: S4 Fig — (A) Left: wild-type disc stained with P-Ser83. Right: disc in which apoptosis has been induced with the driver ptc-Gal4 UAS-rpr (ptc>rpr) and stained with P-Ser83 (n = 5). (B) SalE/Pv>hid discs stained with P-Thr (n = 12) and P-Ser83 (n = 5). YP-1: YO-PRO-1 nuclear staining. Purple: salE/Pv>rpr ablated zone. (C) Staining of Ask1 P-Ser83 after physical injury, (n = 6). Square: magnified image. Scale bars: 50μm. (TIF) [file pgen.1007926.s004.tif]

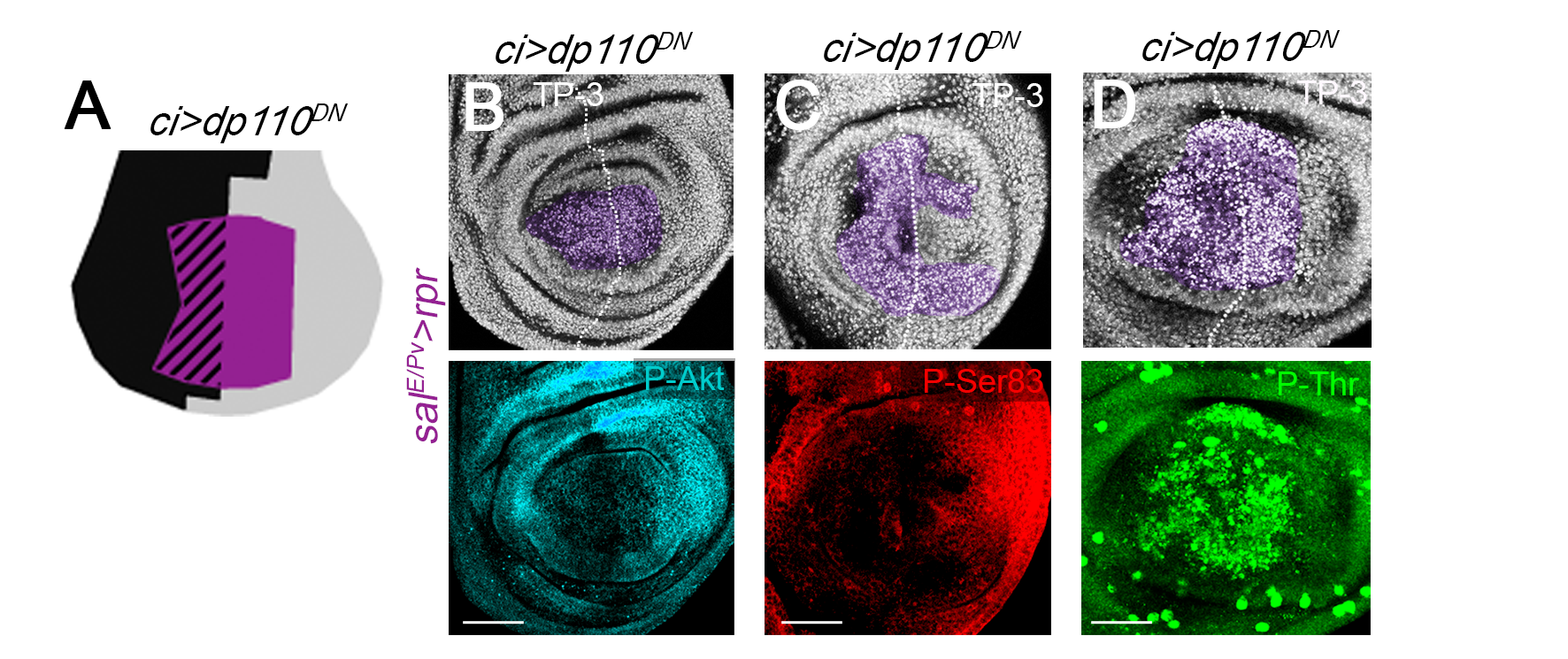

Supplement: S5 Fig — (A) Design of the experiments in B, C and D with salE/Pv>rpr cell death (purple) and blocking Pi3K (ci>dp110DN) in the anterior compartment (black). (B-D) Staining of P-Akt (B), Ask1 P-Ser83 (C) and Ask1 P-Thr (D). White-dotted line indicates anterior-posterior boundary. TP3: TO-PRO-3 nuclear staining. Scale bars: 50μm. (TIF) [file pgen.1007926.s005.tif]

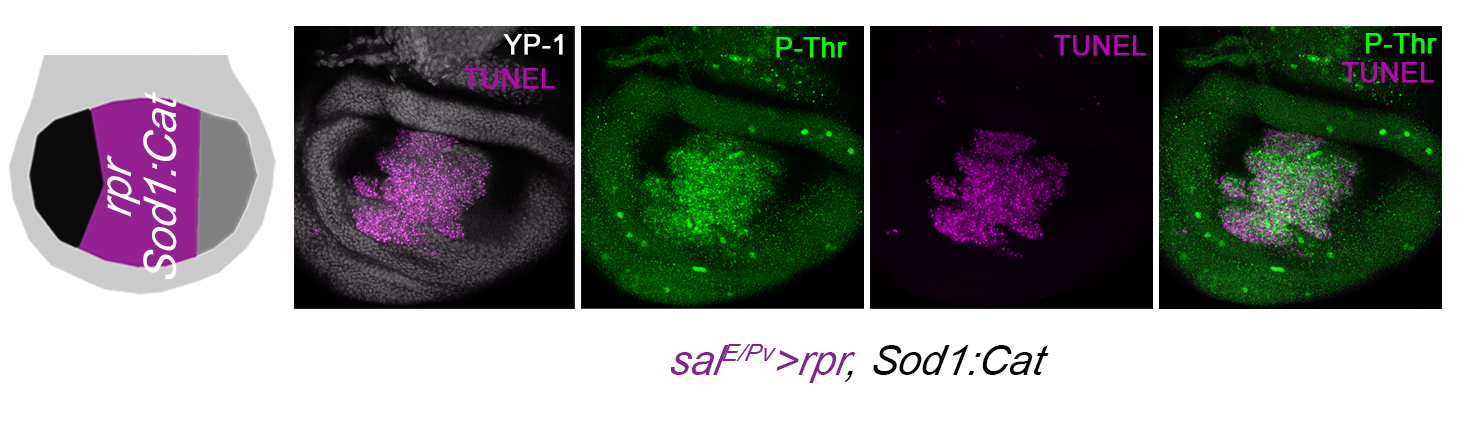

Supplement: S6 Fig — Purple area shows where transgenes were activated. (TIF) [file pgen.1007926.s006.tif]

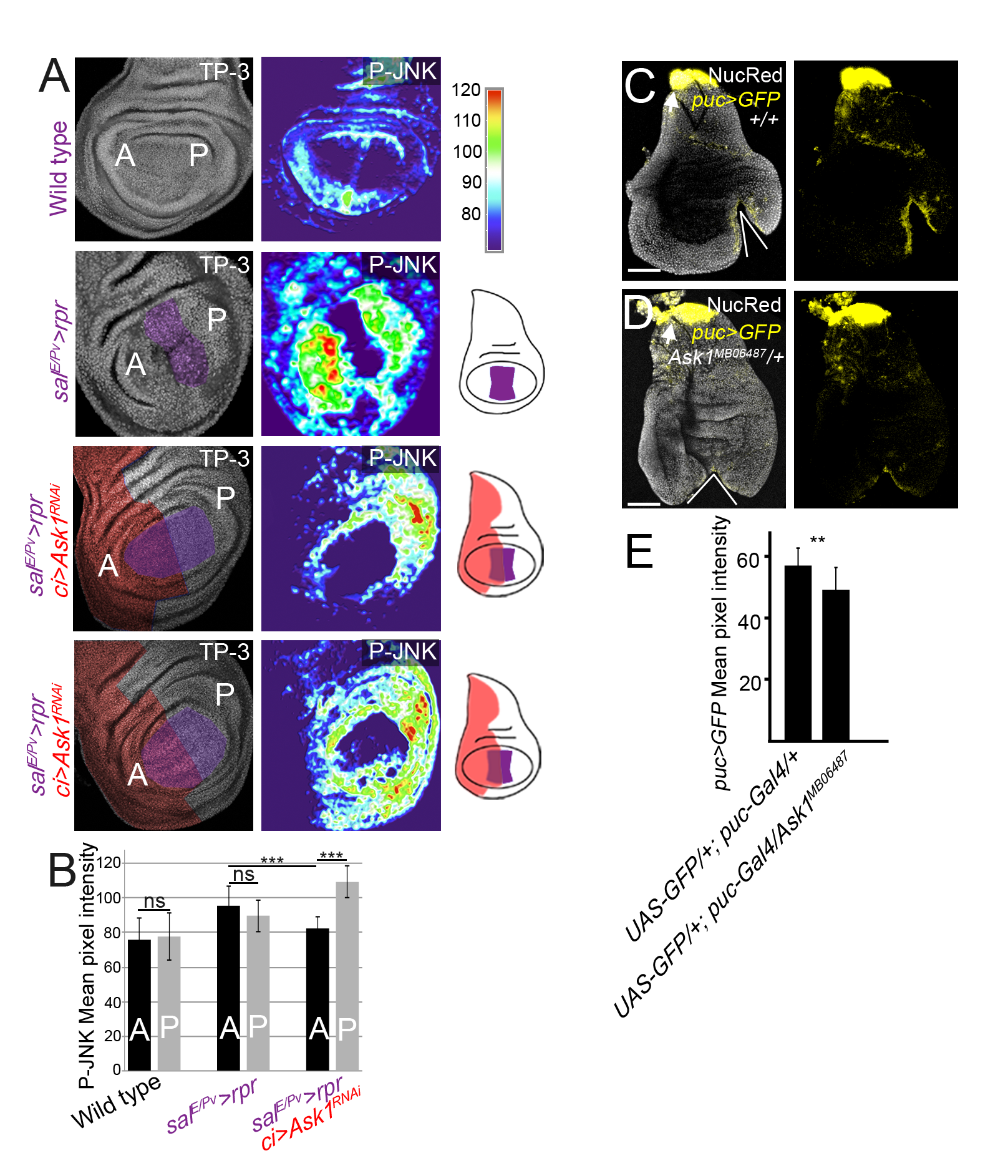

Supplement: S7 Fig — (A) P-JNK staining in wild type, salE/Pv>rpr, and two examples of salE/Pv>rpr ci>Ask1RNAi. Thermal LUT was used to detect pixel differences. TP-3: TO-PRO-3 nuclear staining. Purple indicates the dead zone. Colorized red indicates the ci>Ask1RNAi zone. (B) Quantification of the P-JNK in raw images. A and P indicate the approximate areas measured for pixel intensity in n = 15 for each genotype. (C-E) Live imaging of a puc-Gal4, UAS-GFP used as a reporter of the JNK pathway. Discs were cut and cultured for 6 hours. White lines indicate the wound site. White arrows point to the endogenous puc expression zone at the tip of the notum. (C) Wild type. (D) Ask1MB06487 heterozygous. The nuclear marker NucRed Live647 was added in the culture medium. Scale bar: 50μm. (E) Quantification of puc-Gal4, UAS-GFP reporter at the wound site (n = 13 discs each genotype). Error bars indicate standard deviation. **P<0.01, ***P<0.001. (TIF) [file pgen.1007926.s007.tif]
