# Supplementary material for: Ask1 and Akt act synergistically to promote ROS-dependent regeneration in Drosophila
Source: PLoS Genet. 2019 Jan 24;15(1):e1007926. doi: 10.1371/journal.pgen.1007926 (PMC6363233; doi:10.1371/journal.pgen.1007926)
Supplement: S1 Appendix — This file contains the plain text genewise alignment of the human M3K5_HUMAN protein sequence against the genomic segment of 5kbp upstream/downstream that is centered on the annotated fly Ask1 gene transcription start site. No alignment was reported for the first 126 residues of the human protein where the Ser83 RxRxx[ST] domain has been described. (PDF) [file pgen.1007926.s008.pdf]

Computational verification that human Ser83 domain is missing on fly Ask1 gene loci.

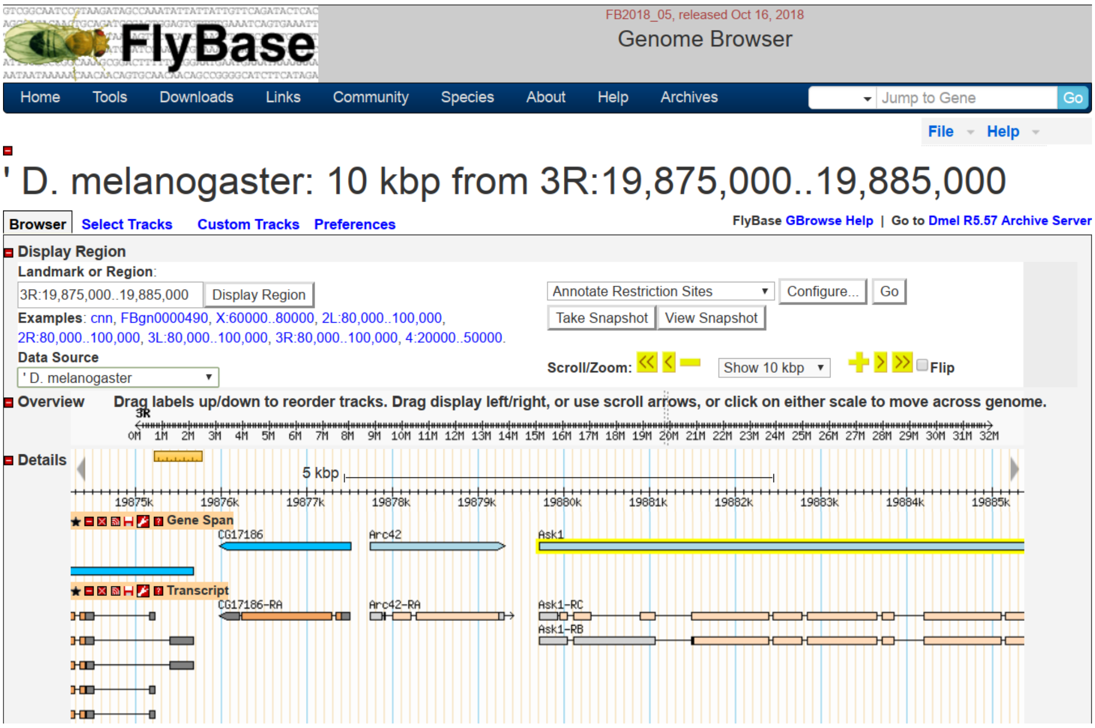

This file contains the plain text genewise alignment of the human M3K5_HUMAN protein sequence against the genomic segment of 5kbp upstream/downstream that is centered on the annotated *Drosophila melanogaster* Ask1 gene transcription start site (see above figure). The genomic sequence segment was retrieved directly from FlyBase (`dm6_chr3R:19,875,000-19,885,000 [+]`).

No alignment was reported for the first 126 residues of the human protein where the Ser83 *RxRxx[ST]* domain has been described. The output from genewise mapping is listed below:

```
genewise output
Score 793.88 bits over entire alignment
Scores as bits over a synchronous coding model

Warning: The bits scores is not probablistically correct for single seqs
See WWW help for more info

sp|Q99683|M3K5_HUMAN  127 LETLHFGKLDFGETTVLDRFYNADIAVVEMSDAFRQPSLFYHLGVRESF
                          L +L F +LDFGET VL+ FYNAD+A++++S   +Q  L YH GVRESF
                          LASLQFERLDFGETNVLETFYNADVAIIDLSILTQQRPLSYHYGVRESF
dm6_chr3R            5102 tgttctgccgtggaagcgattaggggaagcaacaccccttctggagat
                          tcctatagtatgacattactaacatcttatgttcaagctcaaagtgagt
                          gacggcgcgcccggtggggtccctgctccgctccggggtctccctggcc


sp|Q99683|M3K5_HUMAN  176 SMANNIILYCDTNSDSLQSLK---EIICQKN
                           M  NI+ Y D +S    SLK   E++
                          GMKENILTYNDIDSKQTLSLKVRSEVFTWPG
dm6_chr3R            5249 gaagaacatagagtacacacagctggtatcg
                          gtaaattcaaatacaactgtatgcattcgcg
                          ggggcccccccctcgggctcggtgagtcggc


sp|Q99683|M3K5_HUMAN  204                            TMCTGNYTFVPYMITPHNKVYC
                                                       C  NY F+ Y         C
                                                     LSCA-NYLFLSYKRNAETNS-C
dm6_chr3R            5342 GTGCCTG  Intron 1       CAGcttg atctcatacaggaat t
                          <0-----[5342   :   5883]-0>tcgc aatttgaagacacac g
                                                     cgtc ccgtcccggtggctc c
```

```
sp|Q99683|M3K5_HUMAN  226 CDSSFMKGLTELMQPNFELLLGPICLPLVDRFIQLLKVAQASS
                            +S         +PN E   G +   L  R+ + L+  +  S
                          HLTSQPNSGNNSKEPNAE---GRV-PTLQWRLKRKLQDVEIQS
dm6_chr3R            5944 ccaaccaagaaaagcagg   gcg catctccacaccgggact
                          atcgacaggaagaacaca   ggt cctaggtagataatatac
                          cgccgcccccccgactca   aag ctgggccgaggattgcgg


sp|Q99683|M3K5_HUMAN  269                               QYFRESILNDIRKARNLYT
                                                         ++RE +L+D+R AR+ Y
                                     S:K[aaa]           AHMREKFLSDMRTARDTYA
dm6_chr3R            6061 AAGTGAGTA  Intron 2       CAGAgcacgatcagacagcgatg
                            <2-----[6063   :   6484]-2> catgaattgatgccgacac
                                                        ccgtgatgtcgagggcgcc


sp|Q99683|M3K5_HUMAN  289 --GKELAAELARIRQRVDNIEVLTADIVINLLLSYRDIQDYDSIVKLVE
                            G +L + L  +R+R+D+  VL+ ++V + + S RD+QDYD++V+LV
                          TNGAKLQSILHEMRKRLDDPHVLSGEVVHSFMCSLRDVQDYDAMVRLVN
dm6_chr3R            6543 aaggaccaatcgacaccggccgctggggcatattcaggcgtggagccga
                          cagcatagttaatgagtaacattcgattagttgctgataaaacttgtta
                          ccccgggtagcggagggctgtcgtcagactcgcgggtggcctcggagct


sp|Q99683|M3K5_HUMAN  336 TLEKLP-TFDLASHHHVKFHYAFALNRRNLPGDRAKALDIMIPMVQSEG
                           L+ +P T       ++ F YAFALNRRN  GDR KAL+  +  ++ +
                          DLKNIPNTRKYVETGNMSFLYAFALNRRNRKGDREKALESSLKALEKKE
dm6_chr3R            6690 gcaaacaacatggagaaatctgtgcacaacaggcgagcgttcagcgaag
                          ataatcacgaatacgatgttactctaggagagagaactacctactaaaa
                          cggcacccggcgggccgtcgccccgcaatagactagtgatgggggaggg


sp|Q99683|M3K5_HUMAN  384 QVASDMYCLVGRIYKDMFLDSNFTDTESRDHGASWFKKAFESEPTLQSG
                              DM CL GRIYKD+F++S++TD  S  H   W++++FE +P   +G
                          NHFPDMLCLCGRIYKDIFVESDYTDATSLAHAIKWYRQSFEVQPNEYAG
dm6_chr3R            6837 actcgactctgcatagatggtgtaggaatgcgaattccatggccagtgg
                          aatcattgtgggtaaatttacaacaccgtcactagagagtatacaaacg
                          cctgcgccgcccccgcctgacccgccctggcccggccactggggtgtac


sp|Q99683|M3K5_HUMAN  433 INYAVLLLAAGHQFESSFELRKVGVKLSSLLGKKGNLEKLQSYWEVGFF
                          IN A LL+  G +F ++ EL+ +G+ L++L+GKKG+L  L  YW+V  F
                          INLATLLVIEGKEFTNTEELQHIGMTLNNLIGKKGSLSSLSEYWDVATF
dm6_chr3R            6984 aacgaccgaggagtaaaggtccagaataatagaagacttcagttgggat
                          tatccttttagaatcacaataatgtctaattgaaggtcctgaagatcct
                          ctgttggtcgcggtctgggggcccgcactactagccacggtgtgtgccc


sp|Q99683|M3K5_HUMAN  482 LGASVLANDHMRVIQASEKLFKLKTPAWYLKSIVETILIYKHFVKLTTE
                             SVLAND+ + IQA+E +FKLK P WYLKS +  I +   F K   E
                          FEISVLANDYAKAIQAAECMFKLKPPNWYLKSTIGNISLIHRFRKKPEE
dm6_chr3R            7131 tgatgtgagtgagacgggtatacaccattcataagaatcacctcaacgg
                          tatcttcaaacactaccagttataccagatacctgatcttagtgaacaa
                          tgcctgcccccggtatcgcgcgagaccgtgggccatcttatataggcga


sp|Q99683|M3K5_HUMAN  531 -QPVAKQELVDFWMDFLVEATKTD--VTVVRFPVL
                           QP  ++++  FWMDF +EAT T+     +RFP+L
                          RQPPIEEQVFQFWMDFFLEATNTEEVKNSIRFPIL
dm6_chr3R            7278 ccccaggcgtcttagttcggaaagggaaaactcac
                          gacctaaattatgtatttaccacaataagtgtctt
                          cggccggggcgcggcctggccccggtgcctctgtg
```

```
sp|Q99683|M3K5_HUMAN  563                            ILEPTKIYQPSYLSINNEVEEK
                                                     ILEP KIY PSY++IN + +EK
                                                     ILEPQKIYMPSYVTINMDADEK
dm6_chr3R            7383 GTTGGTA  Intron 3       CAGacgccaatacatgaaaaggga
                          <0-----[7383   :   7438]-0>ttacaatatcgatctatacaaa
                                                     ccgtggttgcttgtacgtgtgg


sp|Q99683|M3K5_HUMAN  585 TISIWHV-LPDDK---KGIHEWNFSASSVRGVSISKFEERCCFLYVLHN
                          +I I ++ L   K    K IH++ F AS ++ VS+ K ++RC +LYV HN
                          SIQIVNICLAHAKNACKKIHDFLFVASKIKSVSLYKRDDRCAYLYVHHN
dm6_chr3R            7505 tacagaattgcgaagtaaacgtctggtaaatgactaaggctgtttgcca
                          ctattatgtcacaacgaataattttccatactgtaagaaggcatataaa
                          ctgtgcctgtcagtgtgacccctcgaggcgccctcggttttccgcctcc


sp|Q99683|M3K5_HUMAN  630 SDDFQIYFCTELHCKKFFEMVNTITEEKGRSTEEGDCESDLLE
                          SDDFQIYF +    +KF++M+  +T ++       + ++++ E
                          SDDFQIYFPSTERRQKFYDMILEMTADQVVFVNLSNDDANI-E
dm6_chr3R            7652 tggtcattctagcccattgaacgaaggcggtgacaagggaa g
                          caatatatcccaggaataatttatccaattttatgaaacat a
                          atttaattcctaaaagcctgcgggtatgggtctccttcttc g


sp|Q99683|M3K5_HUMAN  673                            YDYEYDENGDRVVLGKGTYGIV
                                                     Y+Y+YDE   ++VLGKGTYG V
                                                     YEYDYDEQNRKMVLGKGTYGTV
dm6_chr3R            7778 GTAAGTT  Intron 4       TAGtgtgtggcacaagcgagatgag
                          <0-----[7778   :   7843]-0>aaaaaaaaagatttgagcagct
                                                     cgccctggtcagggcatttccg


sp|Q99683|M3K5_HUMAN  695 YAGRDLSNQVRIAIKEIPERDSRYSQPLHEEIALHKHLKHKNIVQYLGS
                          YA RD   QVRIAIKE+PE++S+  QPLHEEI LH  L+H+NIV+YLGS
                          YAARDKQTQVRIAIKEVPEKNSQDVQPLHEEIKLHSQLRHRNIVRYLGS
dm6_chr3R            7910 tggagacacgcagaaggcgaatcggccccggaatctcccccaagcttgt
                          accgaaacatgtctaatcaaacaatactaaatatacatgagattgatgc
                          cccgcgggggacctggggagcggtggagcggtgactggatccatctgag


sp|Q99683|M3K5_HUMAN  744 FSENGFIKIFMEQVPGGSLSALLRSKWGPLKDNEQTIGFYTKQILEGLK
                           SENGF+KIFMEQVPGGSLS LL +KWGPLKDNE T+ FY+KQILEGLK
                          CSENGFFKIFMEQVPGGSLSDLLETKWGPLKDNESTMAFYSKQILEGLK
dm6_chr3R            8057 ttgagttaatagcgcggtctgctgaatgccagagtaagtttacacggca
                          gcaagttatttaatcggctcattacaggctaaaacctctacaattagta
                          cggccccgccgggggaagtcccagcagtgcgccgccgccccggccgggg


sp|Q99683|M3K5_HUMAN  793 YLHDNQIVHRDIKGDNVLINTYSGVLKISDFGTSKRLAGINPCTETFTG
                          YLH+  IVHRDIKGDNVL+NTYSGV+KISDFGTSKRLA INP TETFTG
                          YLHEQDIVHRDIKGDNVLVNTYSGVVKISDFGTSKRLARINPMTETFTG
dm6_chr3R            8204 tccgcgagccgaaggagcgaatagggaatgtgataccgcaacaagatag
                          ataaaattagatagaatttacaggttatcatgccagtcgtactcactcg
                          ctcggctttctagtccggtcgtccgtgcctccccgggccccggggctgc


sp|Q99683|M3K5_HUMAN  842 TLQYMAPEIIDKGPRGYGKAADIWSLGCTIIEMATGKPPFYELGEPQAA
                          TLQYMAPE+ID+G RGYG AADIWS GCT +EMATGKPPF ELG   AA
                          TLQYMAPEVIDQGVRGYGPAADIWSFGCTNVEMATGKPPFIELGSAHAA
dm6_chr3R            8351 atctagcggagcggcgtgcgggatttgtaaggagagacctagcgagcgg
                          ctaatccattaagtggagcccatgctggcatatccgaccttatggcacc
                          cggcgggggtcgaacctcggtccgctacactggtgcaagtcggtcctgc


sp|Q99683|M3K5_HUMAN  891 MFKVGMFKVHPEIPESMSAEAKAFILKCFEPDPDKRACANDLLVDEFLK
                          MFKVG++K HP IPE +SA AK FIL+CF       R  A+ LL D FL+
                          MFKVGFYKKHPNIPEELSANAKNFILRCFAISVMDRPSASQLLEDPFLQ
dm6_chr3R            8498 ataggttaaccaacggctgagaatatcttgaagagcctgtcccggctcc
                          ttatgtaaaacatcaatccacaatttggtctgttagccccattaactta
                          gcggctcggtgcacgggcccagcttgtctcccggcacccgatgatccaa
```

```
sp|Q99683|M3K5_HUMAN  940                               SSKKKKTQPKLSALSAGSN
                                                          K +K +P L
                                     V:D[gat]           --KPRKVRPALPI----NT
dm6_chr3R            8645 GAGTGAGTT  Intron 5       AAGT  accagccgcca    aa
                            <2-----[8647   :   8709]-2>   acgatgcctct    ac
                                                          accggcacgcc    tt


sp|Q99683|M3K5_HUMAN  960 EYLRSISLPVPVLVEDTSSSSEYGSV--SPDTELK
                          E+ RSIS+P    V  T+    Y +    P T
                          EFGRSISVPADRFVHKTTPPLSYNTTCNTPTTPEL
dm6_chr3R            8750 gtgcaatgcggctgcaaacccataaataacaacgt
                          atgggtctccagttaacccctgaaccgacccccat
                          gcacctgggttgtgcacagggcccacctgcaggag


sp|Q99683|M3K5_HUMAN  993                               DPFSFKTRAKSCGERDVKG
                                                        + F ++ R  S G
                                     V:D[gac]           NQFMLERR-NSHG------
dm6_chr3R            8855 GAGTAAGTT  Intron 6       CAGCactacgcc atcg
                            <2-----[8857   :   9238]-2> aatttagg acag
                                                        cgtgtaga tcta


sp|Q99683|M3K5_HUMAN 1013 IRTLFLGIPDENFEDHSAPPSPEE--KDSGFFMLRKDSERRATLHRILT
                              FL  P+      S   S  E     GF+ L+KDS+RR TL ++L
                          ----FLLSPEIEPSTPSLRTSISETSETDGFYRLKKDSQRRTTLSKVLA
dm6_chr3R            9276     ttctcgagctacttcaaaagaagaggttacaagtcacaactagcg
                              tttccatacccccctgcgtgacgacagtagtaaacaggcctcattc
                              tgcgagtatcggcgtgcttggcgtcttcgtagtggggagtcggga


sp|Q99683|M3K5_HUMAN 1060 EDQDKIVRNLMESLAQGAEEPKLKWEHITTLIASLREFVRSTDRKIIAT
                           D+ KI    ++ +        ++   +  LI  LR+++ + + K +
                          LDESKICDIWLKKVDADQNSIAIRKSDLEVLIRGLRDYIMNENEKHLEA
dm6_chr3R            9411 cggtaatgatcaaggggcatagaaaagtggcacgccgtaaagagactgg
                          taacatgatgtaatacaaactctgagatatttggtgaattaaaaaatac
                          acacgccttggggccttgcgtgtgactggtacaaggcctgcacagtgag


sp|Q99683|M3K5_HUMAN 1109 TLSKLKLELDFDSHGISQVQVVLFGFQDAVNKVLRNHNIKPHWMFALDS
                          T+++LK +L+ D+  +  + + L+ FQDAV  VLR H IKPHWMFALD+
                          TINELKQKLNNDAVALDHLHLALYSFQDAVVCVLRYHCIKPHWMFALDN
dm6_chr3R            9558 aaagcacacaaggggcgccctgctttcggggtgcctctaacctatgtga
                          ctaataaataaactctaatatctactaacttgttgaagtacagttctaa
                          tccagaaggcccattgctttgggcctgccgccaggctccactggcagtt


sp|Q99683|M3K5_HUMAN 1158 IIRKAVQTAITILVPEL
                          ++++AVQ A+TI  PEL
                          LVKRAVQAAVTIFSPEL
dm6_chr3R            9705 cgacggcgggaattcgc
                          ttagctacctcttccat
                          gtggccgggcttcttag


//
```
