# Supplementary material for: Ask1 and Akt act synergistically to promote ROS-dependent regeneration in Drosophila
Source: PLoS Genet. 2019 Jan 24;15(1):e1007926. doi: 10.1371/journal.pgen.1007926 (PMC6363233; doi:10.1371/journal.pgen.1007926)
Supplement: S2 Appendix — Many positions are preserved from sponges to humans, including serine/threonine residues, whilst other show a dual pattern of conservation with specific substitutions between chordate and invertebrate species. From the whole set of 338 sequences on 164 species that define the EggNOG (http://eggnogdb.embl.de) homologs cluster KOG4279, comprising human MAP3K5 and its counterpart in fly Ask1-PC, we filtered out 49 proteins that were confidently annotated as MAP3K5 orthologs on different taxa plus fly Ask1-PB isoform downloaded from FlyBase, which are listed here (names shown in parentheses are those used on the alignment sequence labels and refer to UniProt identifiers when possible): Homo sapiens ENSP00000351908 (M3K5_HUMAN), Pan troglodytes ENSPTRP00000043317 (K7CFQ9_PANTR), Gorilla gorilla ENSGGOP00000022651 (G3S3K1_GORGO), Macaca mulatta ENSMMUP00000021508 (F7G2W1_MACMU), Pongo abelii ENSPPYP00000019077 (H2PKF0_PONAB), Otolemur garnettii ENSOGAP00000000498 (H0WGN9_OTOGA), Callithrix jacchus ENSCJAP00000015659 (F7GZH9_CALJA), Rattus norvegicus ENSRNOP00000051338 (D3ZW27_RAT), Mus musculus ENSMUSP00000093485 (M3K5_MOUSE), Cavia porcellus ENSCPOP00000003829 (H0V2Q4_CAVPO), Bos taurus ENSBTAP00000011949 (F1MXH6_BOVIN), Pteropus vampyrus ENSPVAP00000009127 (ens9127_PTEVA), Mustela putorius furo ENSMPUP00000014726 (G9K9I6_MUSPF), Loxodonta africana ENSLAFP00000000494 (G3UDL3_LOXAF), Echinops telfairi ENSETEP00000012543 (ens12543_ECHTE), Canis lupus familiaris ENSCAFP00000000362 (F1PA02_CANFA), Ailuropoda melanoleuca ENSAMEP00000010579 (D2H0V2_AILME), Tursiops truncatus ENSTTRP00000002388 (ens2388_TURTR), Dasypus novemcinctus ENSDNOP00000014966 (ens14966_DASNO), Sarcophilus harrisii ENSSHAP00000014955 (G3WHQ0_SARHA), Gallus gallus ENSGALP00000022482 (F1NYS9_CHICK), Meleagris gallopavo ENSMGAP00000013731 (G1NK21_MELGA), Taeniopygia guttata ENSTGUP00000011202 (H0ZKT1_TAEGU), Pelodiscus sinensis ENSPSIP00000018784 (K7GES3_PELSI), Xenopus tropicalis ENSXETP00000005271 (F7A415 [file pgen.1007926.s009.pdf]

Alignment: M3K5human_eggnog.selection.mafft.aln
Seaview [blocks=10 fontsize=5 A4-landscape] on Thu Nov 29 16:50:16 2018

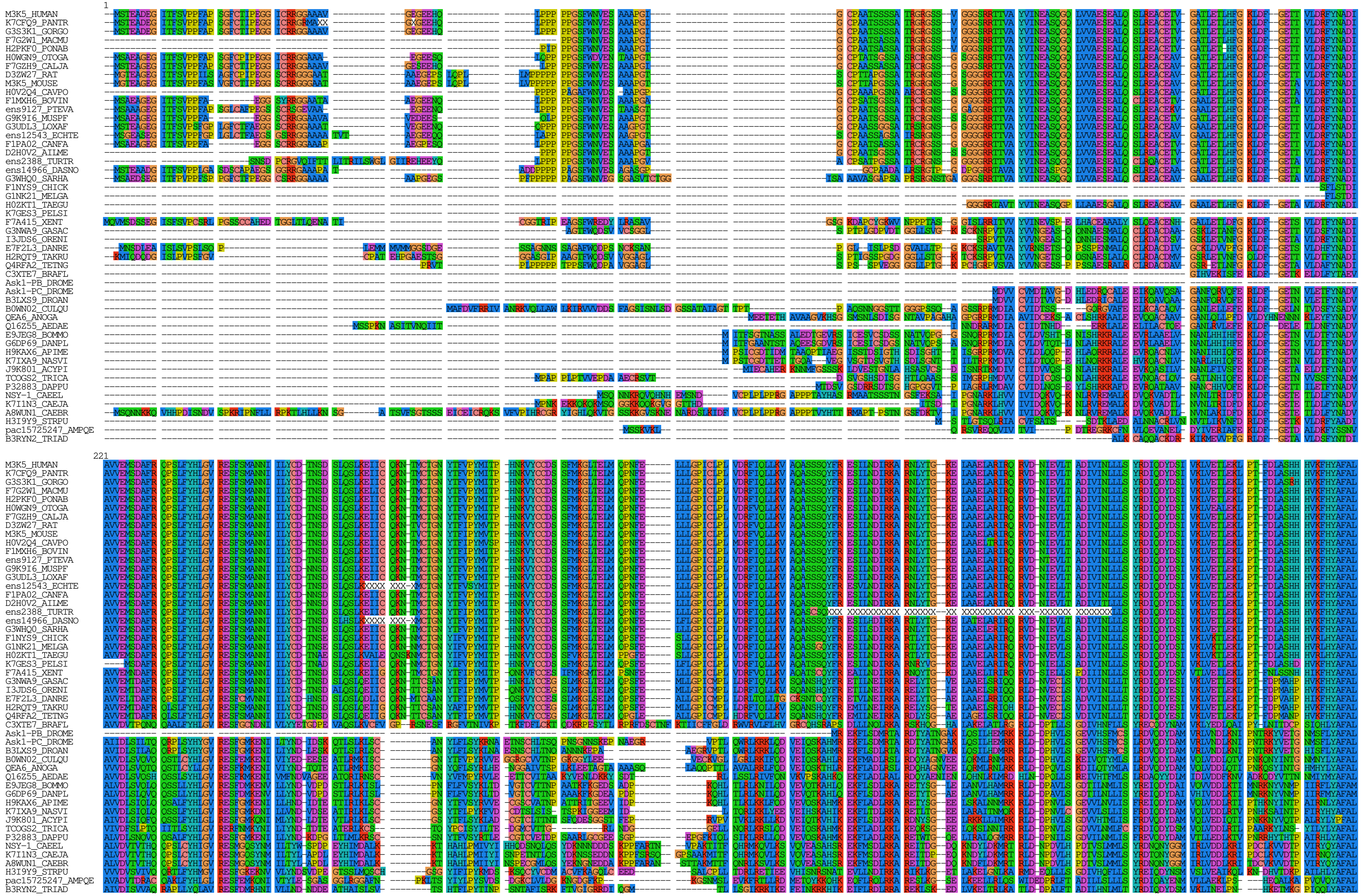

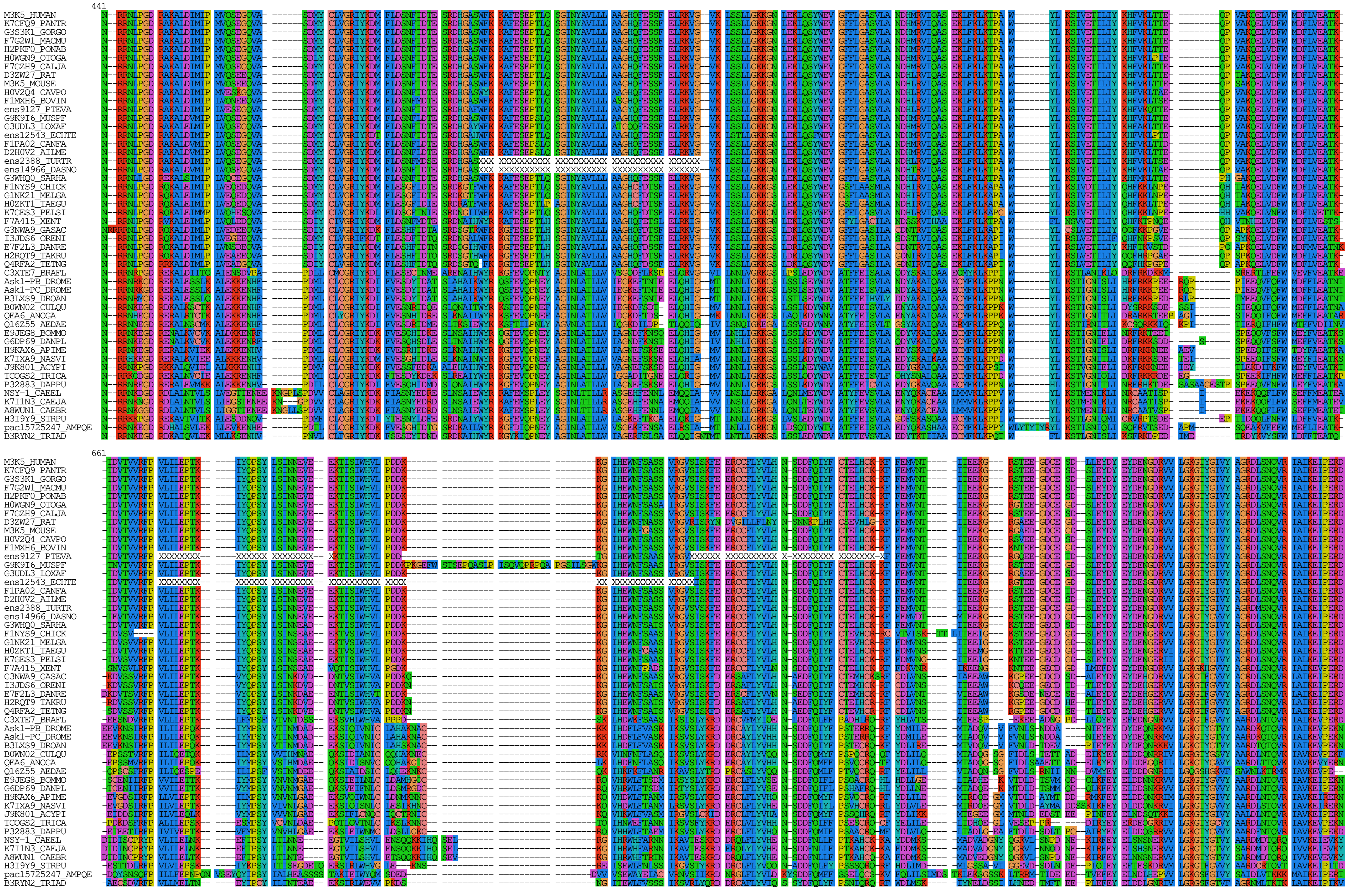
441
661
M3K5_HUMAN
K7CFQ9_PANTR
G3S3K1_GORGO
F7G2W1_MACMU
H2PKF0_PONAB
H0WGN9_OTOGA
F7GZH9_CALJA
D3ZW27_RAT
M3K5_MOUSE
H0V2Q4_CAVPO
F1MXH6_BOVIN
ens9127_PTEVA
G9K9I6_MUSPF
G3UDL3_LOXAF
ens12543_ECHTE
F1PA02_CANFA
D2H0V2_AILME
ens2388_TURTR
ens14966_DASNO
G3WHQ0_SARHA
F1NYS9_CHICK
G1NK21_MELGA
H0ZKT1_TAEGU
K7GES3_PELSI
F7A415_XENT
G3NWA9_GASAC
I3JDS6_ORENI
E7F2L3_DANRE
H2RQT9_TAKRU
Q4RFA2_TETNG
C3XTE7_BRAFL
Ask1-PB_DROME
Ask1-PC_DROME
B3LXS9_DROAN
B0WN02_CULQU
Q0EA6_ANOGA
Q16Z55_AEDAE
E9JEG8_BOMMO
G6DP69_DANPL
H9KAX6_APIME
K7IXA9_NASVI
J9K801_ACYPI
TCOGS2_TRICA
P32883_DAPPU
NSY-1_CAEEL
K7I1N3_CAEJA
A8WUN1_CAEBR
H3I9Y9_STRPU
pac15725247_AMPQE
B3RYN2_TRIAD

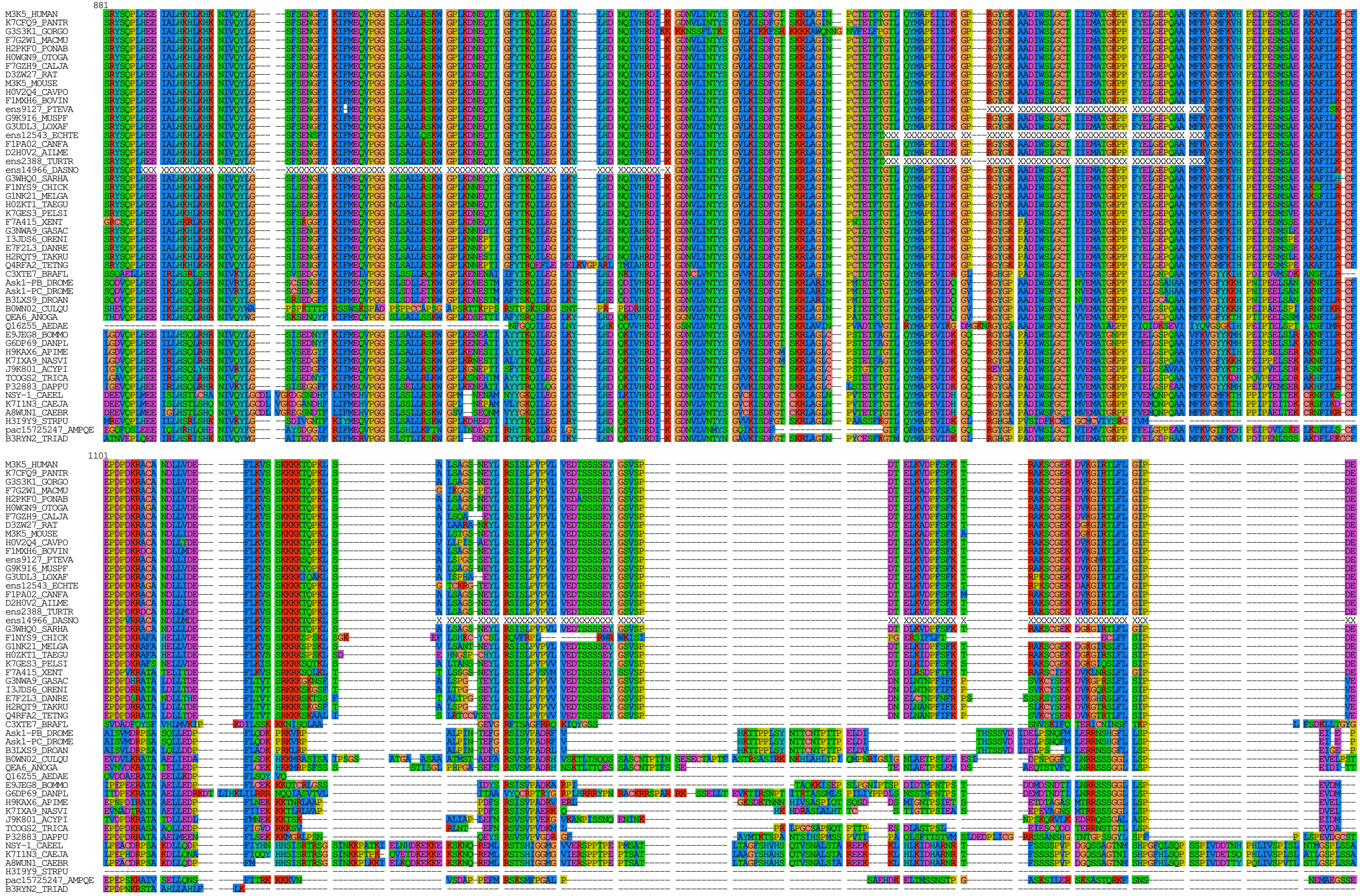
881
M3K5_HUMAN
K7CFQ9_PANTR
G3S3K1_GORGO
F7G2W1_MACMU
H2PKF0_PONAB
H0WGN9_OTOGA
F7GZH9_CALJA
D3ZW27_RAT
M3K5_MOUSE
H0V2Q4_CAVPO
F1MXH6_BOVIN
ens9127_PTEVA
G9K9I6_MUSPF
G3UDL3_LOXAF
ens12543_ECHTE
F1PA02_CANFA
D2H0V2_AILME
ens2388_TURTR
ens14966_DASNO
G3WHQ0_SARHA
F1NYS9_CHICK
G1NK21_MELGA
H0ZKT1_TAEGU
K7GES3_PELSI
F7A415_XENT
G3NWA9_GASAC
I3JDS6_ORENI
E7F2L3_DANRE
H2RQT9_TAKRU
Q4RFA2_TETNG
C3XTE7_BRAFL
Ask1-PB_DROME
Ask1-PC_DROME
B3LXS9_DROAN
B0WN02_CULQU
Q0EA6_ANOGA
Q16255_AEDAE
E9JEG8_BOMMO
G6DP69_DANPL
H9KAX6_APIME
K7IXA9_NASVI
J9K801_ACYPI
TCOGS2_TRICA
P32883_DAPPU
NSY-1_CAEEL
K7I1N3_CAEJA
A8WUN1_CAEBR
H3I9Y9_STRPU
pac15725247_AMPQE
B3RYN2_TRIAD
1101

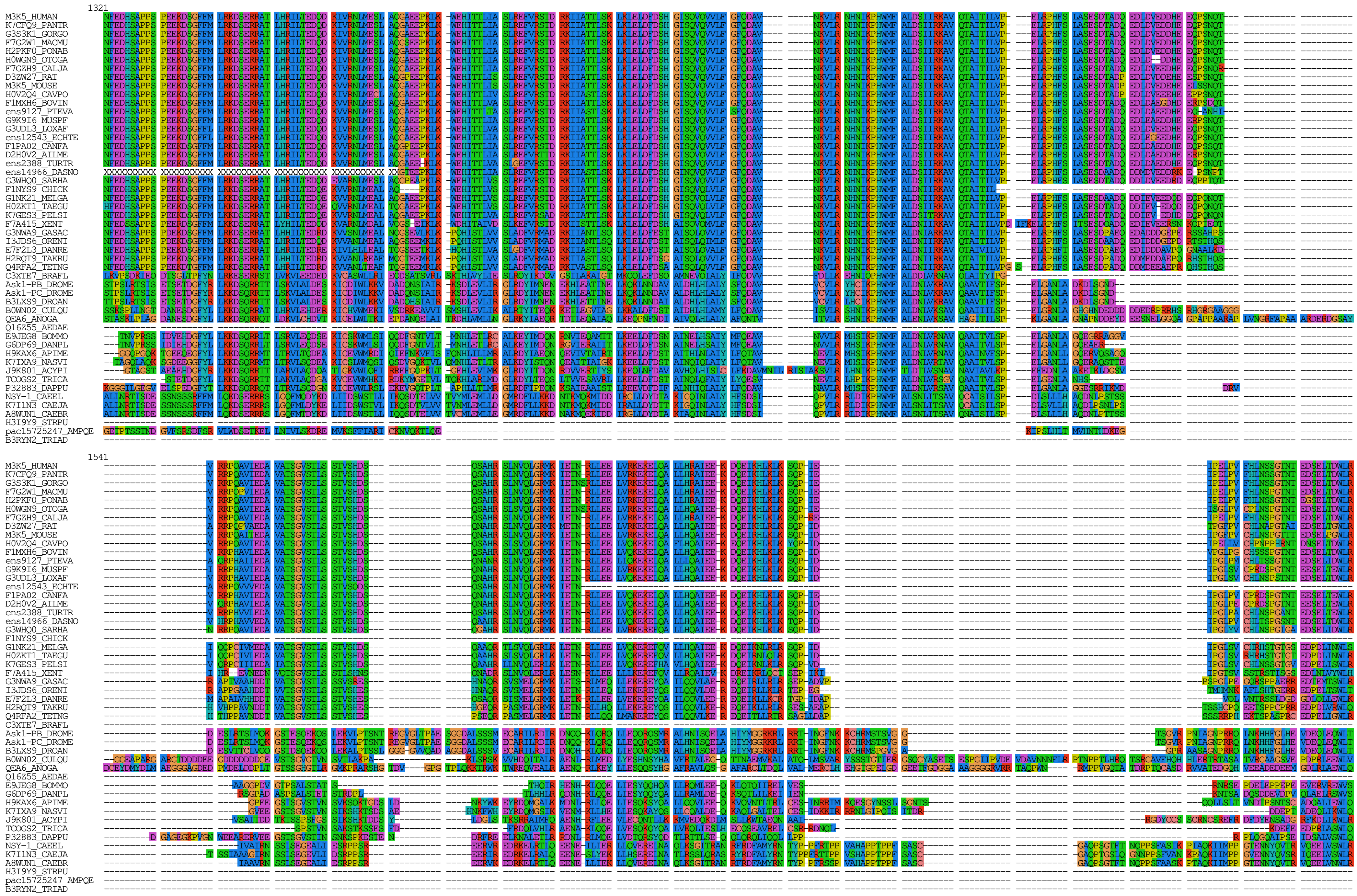
1321
M3K5_HUMAN
K7CFQ9_PANTR
G3S3K1_GORGO
F7G2W1_MACMU
H2PKF0_PONAB
H0WGN9_OTOGA
F7GZH9_CALJA
D3ZW27_RAT
M3K5_MOUSE
H0V2Q4_CAVPO
F1MXH6_BOVIN
ens9127_PTEVA
G9K9I6_MUSPF
G3UDL3_LOXAF
ens12543_ECHTE
F1PA02_CANFA
D2H0V2_AILME
ens2388_TURTR
ens14966_DASNO
G3WHQ0_SARHA
F1NYS9_CHICK
G1NK21_MELGA
H0ZKT1_TAEGU
K7GES3_PELSI
F7A415_XENT
G3NWA9_GASAC
I3JDS6_ORENI
E7F2L3_DANRE
H2RQT9_TAKRU
Q4RFA2_TETNG
C3XTE7_BRAFL
Ask1-PB_DROME
Ask1-PC_DROME
B3LXS9_DROAN
B0WN02_CULQU
Q0EA6_ANOGA
Q16255_AEDAE
E9JEG8_BOMMO
G6DP69_DANPL
H9KAX6_APIME
K7IXA9_NASVI
J9K801_ACYPI
TCOGS2_TRICA
P32883_DAPPU
NSY-1_CAEEL
K7I1N3_CAEJA
A8WUN1_CAEBR
H3I9Y9_STRPU
pac15725247_AMPQE
B3RYN2_TRIAD
1541

```
                  1761
M3K5_HUMAN        VNGADE-DTI SRFLAEDYTL LDVLYYVTRD DLKCLRLRGG MLCTLWKAII DFRNKQT--- ---------- ---------- ---------- ---------- ---------- ---------- --------
K7CFQ9_PANTR      VNGADE-DTI SRFLAEDYTL LDVLYYVTRD DLKCLRLRGG MLCTLWKAII DFRNKQT--- ---------- ---------- ---------- ---------- ---------- ---------- --------
G3S3K1_GORGO      VNGADE-DTI SRFLAEDYTL LDVLYYVTRD DLKCLRLRGG MLCTLWKAII DFRNKQT--- ---------- ---------- ---------- ---------- ---------- ---------- --------
F7G2W1_MACMU      VNGADE-DTI SRFLAEDYTL LDVLYYVTRD DLKCLRLRGG MLCTLWKAII DFRNKQT--- ---------- ---------- ---------- ---------- ---------- ---------- --------
H2PKF0_PONAB      VNGADE-DTI SRFLAEDYTL LDVLYYVTRD DLKCLRLRGG MLCTLWKAII DFRNKQT--- ---------- ---------- ---------- ---------- ---------- ---------- --------
H0WGN9_OTOGA      ENGADE-DTI SRFLAEDYTL DDVLYCVTRD DLKCLRLRGG MLCTLWKAIV DFRDKKT--- ---------- ---------- ---------- ---------- ---------- ---------- --------
F7GZH9_CALJA      ENGADE-DTI SRVLAEDYTL VDVLYYVTRD DLKCLRLRGG MLCTLWKAII DFRNKQT--- ---------- ---------- ---------- ---------- ---------- ---------- --------
D3ZW27_RAT        ENGADE-DTI SRFLAEDYTL VDVLYYVTRD DLKCLRLRGG MLCTLWKAII DFRNKG---- ---------- ---------- ---------- ---------- ---------- ---------- --------
M3K5_MOUSE        ENGADE-DTI SRFLAEDYTL VDVLYYVTRD DLKCLRLRGG MLCTLWKAII DFRNKC---- ---------- ---------- ---------- ---------- ---------- ---------- --------
H0V2Q4_CAVPO      QNGADE-DTI SRFLDEKYTL VDVLYYVTRD DLKYLRLRGG MLCTLWKAII DFRNK----- ---------- ---------- ---------- ---------- ---------- ---------- --------
F1MXH6_BOVIN      ENGADE-DTI SRFLAEDYSL MDVLYYVTRD DLKCLRLRGG MLCTLWKAIT DFRDKQA--- ---------- ---------- ---------- ---------- ---------- ---------- --------
ens9127_PTEVA     ENGADE-DTI SRFLAEDFSL VDVLYYATRD DLKCLRLRGG MLCTLWKAII NYREKQAE-- ---------- ---------- ---------- ---------- ---------- ---------- --------
G9K9I6_MUSPF      DKGADE-DTI SRFLAEDYSL EDVLYYVTRD DLKCLRLRGG MLCALWKAIT DFRDTQT--- ---------- ---------- ---------- ---------- ---------- ---------- --------
G3UDL3_LOXAF      ENGADE-DTI SRFLAEDYTL VDVLYYVTRD DLKCLRLRGG MLCTLWKAIV DFRDK----- ---------- ---------- ---------- ---------- ---------- ---------- --------
ens12543_ECHTE    ---------- ---------- ---------- ---------- ---------- ---------- ---------- ---------- ---------- ---------- ---------- ---------- --------
F1PA02_CANFA      DKGADE-DTI SRFLAEDYSL EDVLYYVTRD DLKCLRLRGG MLCTLWKAIT DFRDTQA--- ---------- ---------- ---------- ---------- ---------- ---------- --------
D2H0V2_AILME      EKGADE-DTI SR-------- ---------- ---------- ---------- ---------- ---------- ---------- ---------- ---------- ---------- ---------- --------
ens2388_TURTR     ENGADE-DTI SRFLAEDYSL ADVLYYVTRD DLKCLRLRGG MLCTLWKAIT DFRDKQT--- ---------- ---------- ---------- ---------- ---------- ---------- --------
ens14966_DASNO    ENGADE-DTI SRXXXXXXXX XXXXXXXXXX XXXXXXXXGG VLCSLWKAIT DFRDKRF--- ---------- ---------- ---------- ---------- ---------- ---------- --------
G3WHQ0_SARHA      ENGADE-DTI SRFLAEDYTL VDVLYCVTRD DLKCLRLRGG MLCTLWKAIS DFREKKP--- ---------- ---------- ---------- ---------- ---------- ---------- --------
F1NYS9_CHICK      ---------- ---------- ---------- ---------- ---------- ---------- ---------- ---------- ---------- ---------- ---------- ---------- --------
G1NK21_MELGA      VQGADE-ATI NKFLAEDYTL MDVLCYVTRD DLKCLRLRGG MLCTLWKAIT DFRQKHA--- ---------- ---------- ---------- ---------- ---------- ---------- --------
H0ZKT1_TAEGU      VHGADE-ATI NKFLAEDYTL TDVLCYVTRD DLKCLRLRGG MLCTLWKAIT EFREKH---- ---------- ---------- ---------- ---------- ---------- ---------- --------
K7GES3_PELSI      VHGADE-ETI NRFLAEDYTL MDVLCYVTRD DLKCLRLRGG MLCMLWKAIS DFREKRA--- ---------- ---------- ---------- ---------- ---------- ---------- --------
F7A415_XENT       KCRVDE-DTI SKFLAEDYQL EDILCHVTRD DLKSLNLRGG VLCTIWKEIT DYREKQA--- ---------- ---------- ---------- ---------- ---------- ---------- --------
G3NWA9_GASAC      LYGADQ-DSV DRILNEEYTL NNILHDVTRD DLKGLRLRGG ILCKLWKAIT DYRQKHI--- ---------- ---------- ---------- ---------- ---------- ---------- --------
I3JDS6_ORENI      RYGADQ-DAI DRILSEEYTL NDILHDVTRD DLKSLRLRGG VLCKVWKAIT DYRQKLF--- ---------- ---------- ---------- ---------- ---------- ---------- --------
E7F2L3_DANRE      LHGADA-DSI ERIIAEDYSL DDILHYVTRD DLKTLRLRGG VLCKLWSAIT EHRKKPG--- ---------- ---------- ---------- ---------- ---------- ---------- --------
H2RQT9_TAKRU      LHGADQ-ESI DRILSEEYAL NDILNDVTRD DLKSLRLRGG VLCKLWKAIT EYRQKPT--- ---------- ---------- ---------- ---------- ---------- ---------- --------
Q4RFA2_TETNG      LHGADQ-EAV DRILDEEYTL SDILHDVTRD DLKSLSLRGG VLCKLWKAIT EYRQKP---- ---------- ---------- ---------- ---------- ---------- ---------- --------
C3XTE7_BRAFL      ---------- ---------- ---------- ---------- ---------- ---------- ---------- ---------- ---------- ---------- ---------- ---------- ----GIST
Ask1-PB_DROME     QQEIDE-FSK TLIILNEGFTF DDFIYNMEKL DLMRLGLRVG IEVRLWKLII QERVCTSSDC VDSPPSTYHK PIGGNSSFAL IDNNNTLPSP TTTTPGTP-- GIKTKIKKSE SEYDSCSE-- --------
Ask1-PC_DROME     QQEIDE-FSK TLIILNEGFTF DDFIYNMEKL DLMRLGLRVG IEVRLWKLII QERVCTSSDC VDSPPSTYHK PIGGNSSFAL IDNNNTLPSP TTTTPGTP-- GIKTKIKKSE SEYDSCSE-- --------
B3LXS9_DROAN      QQEIDE-FSK TLIILNEGFTF EDFIYNMEKL DLMRLGLRVG IEVRLWKLII QERACTSPDC LDSPPSTYHK PIGGNGSLAL MTNNNTLPSP TTTTPNTPDT GIKTKIKKSE SEYDSCSE-- --------
B0WN02_CULQU      QHGIGV-AVR NTIHGEEFSF EDFVYGMEQD DLRRIGLRVG TEVKLWRAIR AYRSQHPLPH TTEPAPSLST VLANGDSTPS TDEPQLRRQQ PQHKPISNSN SWDSSHSTAT STSSEYES-- --CNGEGS
QEA6_ANOGA        AQGIKRPECQ RRILREGFLC DDFLYDLERD DLRRLGLKIG EELRIWNALK RHRRLHPPKR PSADCDSVHN INNNCLNDHH TAGLDTR--- ---------- ---------- ---------- --------
Q16Z55_AEDAE      ---------- ---------- ---------- ---------- ---------- ---------- ---------- ---------- ---------- ---------- ---------- ---------- --------
E9JEG8_BOMMO      SRGLPA-AVA RALSRQHYTL TDLLLHAQRE DVSRLHLSGG IELRLWRAIQ EHRLHAGPTH LRRSSSTETD IDTNSIVVVE CQRCKSIRRT TSTNSNCSSN PTVVIREHQV NGESPLTQLY GENGVDNL
G6DP69_DANPL      SCGVPD-EVV RALAAQKYNL DDLLHHAERE DLARLRLKGG VELRLWRAIV EHR--SQDNH LRRTSSTETD ID-NTIVVVE CQKCKTSRSA TT----CSNN PTIVIRDQKN GDD--VAEYY AENGVVNL
H9KAX6_APIME      NLQIDD-MTI ERFLYEQYTL EDILNHVTRD DIRRLNFRGG IELRIWQAIL KHRQSNNE-- ---------- ---------- ---------- ---------- ---------- ---------- --------
K7IXA9_NASVI      SLNIDG-NTI ERFVSEQYTL EDILVYVTRD DLRRLNLKGG IELRIWKAIL ARKKGSTP-- ---------- ---------- ---------- ---------- ---------- ---------- --------
J9K801_ACYPI      DICIDE-HSI KILVNEQFLL DQIQLEVTRE DLRDLGLKRG VELRLWKEIE RHRENNLSSS GGTMTSVTPQ PLLPSSTNNN NYAFDALQQL QSSHRMSN-- ---------- ---------- -HINNNYV
TCOGS2_TRICA      GLGVNE-FGR KKILAEGYTL NEVLYNIGRE ELQRIGLRGA VEFRIWRAIE QHRQSPNVYL CNGVTDDTST V--------- ---------- ---------- ---------- ---------- --------
P32883_DAPPU      SNNVEE-CDI ALFTSEGYIL IDVIEFFTRE DLRRLGLRGG TELRLWRAIL EQRAQSNIT- ---------- ---------- ---------- ---------- ---------- ---------- --------
NSY-1_CAEEL       GLEIDE-RSI ALIASEAYTK SDMMDFVTRD ELLSIGVGGG SSCRIMRAIG EVRERQRRQP VFLSPMRSRD DSLDDYHSSS ADDMYTGAAA ETSSGN---- ---------- ---------- --------
K7I1N3_CAEJA      SLEIDE-RSI ALIASEAYSK NDLIDFVTRD ELLSIGVGGG SSCRIMRAVN EIRERQRRQP VFLSPMRSRD DSLDDYHSSS ADDMFHVAAA EAAT------ ---------- ---------- --------
A8WUN1_CAEBR      GLEIDE-RST ALIASEAYTK NDLIDFVTRD ELLNIGIGGG PSCRIMRAIG EIRERQRRQP VFLSPMRSRD DSLDDYHSSS ADDMFTTVSA ADATP----- ---------- ---------- --------
H3I9Y9_STRPU      ---------- ---------- ---------- ---------- ---------- ---------- ---------- ---------- ---------- ---------- ---------- ---------- --------
pac15725247_AMPQE -----G-GGL FLFLGTP--- ---------- ---------- ---------- ---------- ---------- ---------- ---------- ---------- ---------- ---------- --------
B3RYN2_TRIAD      ---------- ---------- ---------- ---------- ---------- ---------- ---------- ---------- ---------- ---------- ---------- ---------- --------
```
