# Supplementary material for: Ask1 and Akt act synergistically to promote ROS-dependent regeneration in Drosophila
Source: PLoS Genet. 2019 Jan 24;15(1):e1007926. doi: 10.1371/journal.pgen.1007926 (PMC6363233; doi:10.1371/journal.pgen.1007926)
Supplement: S3 Appendix — (DOCX) [file pgen.1007926.s010.docx]

**S3 Appendix. Data for the graphics of figure 4**

Data for figure 4B Mean pixel intensities of P-Thr fluorescent labeling in the apoptotic zone of *sal^E/Pv^>rpr* discs from larvae fed with a standard (dying cells 94.01±17.04; S.D., n=11) or NAC-supplemented (dying cells 31.07±9.38; S.D. n=10) diet.

Data for figure 4C. Mean pixel intensities of Ask1 P-Ser83 fluorescent labeling in the neighboring cells near the apoptotic zone of *sal^E/Pv^>rpr* discs from larvae fed with a standard (living cells 57.56±23.27; S.D., n=20) or NAC-supplemented (living cells 29.54±5.92; S.D., n=18) diet.

Data for figure 4E. Left: P-Thr mean pixel intensity of wild-type wing discs from larvae fed with standard (79.36±18.65; S.D., n=17), H_2_O_2_- (93.37±3.87; S.D., n=7) and tunicamycin-supplemented (125.49±12.51; S.D., n=7) food. Right: P-Thr mean pixel intensity of *Ask1^MB06487^* homozygous mutant discs from larvae fed with standard (92.92±9.75; S.D., n=6), H_2_O_2_- (83.66±7.11; S.D. n=11) and tunicamycin-supplemented (87.99±21.38; S.D., n=7) food.

Data for figure 4F. Left: P-Ser83 mean pixel intensity of wild-type wing discs from larvae fed with standard (79.14±17.69; S.D., n=19), H_2_O_2_- (110.89±15.43; S.D., n=14) and tunicamycin-supplemented (128.35±14.42; S.D., n=5) food.

Right: P-Ser83 mean pixel intensity of *Ask1^MB06487^* homozygous mutant discs from larvae fed with standard (81.83±20.25; S.D., n=12), H_2_O_2_- (87.51±6.40; S.D., n=6) and tunicamycin-supplemented (90.27±13.26; S.D.; n=4) food.

Data for figure 4G. P-Akt mean pixel intensity for *sal^E/Pv^>rpr, GFP* was 100.61±25,14, S.D. in A and 92.64±21.38, S.D. in P; and for *sal^E/Pv^>rpr, Sod1:Cat* was 68,04±18,16, S.D. in A and 63.14±15.66, S.D. in P. P-Ser83 mean pixel intensity for *sal^E/Pv^>rpr, GFP* was 86.89±34.49, S.D. in A and 78.46±31.06, S.D. in P; and for *sal^E/Pv^>rpr, Sod1:Cat* was 63.25±23.82, S.D. in A and 54.32±19.92, S.D. in P. N= 23 discs for each genotype.
